# Effect of Physical Exercise and Genetic Background on Glucose Homeostasis and Liver/Muscle Proteomes in Mice

**DOI:** 10.3390/metabo12020117

**Published:** 2022-01-25

**Authors:** Mileni S. Fernandes, Isabela T. Sabino-Arias, Aline Dionizio, Mayara F. Fabricio, Juliana S. Trevizol, Tatiana Martini, Liane B. Azevedo, Ruth A. Valentine, Anne Maguire, Fatemeh V. Zohoori, Sandra L. Amaral, Marília A. R. Buzalaf

**Affiliations:** 1 Bauru School of Dentistry, University of São Paulo, Bauru 17012-901, SP, Brazil; mi_biol@yahoo.com.br (M.S.F.); belatomazini@gmail.com (I.T.S.-A.); alinesdionizio@usp.br (A.D.); julianatrevizol@usp.br (J.S.T.); tatianamartini@usp.br (T.M.); 2 Department of Physical Education, School of Sciences, São Paulo State University, Bauru 17033-360, SP, Brazil; mayara.fabricio@hotmail.com (M.F.F.); amaral.cardoso@unesp.br (S.L.A.); 3 School of Human and Health Sciences, University of Huddersfield, Huddersfield HD1 3DH, UK; l.azevedo@tees.ac.uk; 4 Centre for Oral Health Research, School of Dental Sciences, Newcastle University, Newcastle upon Tyne NE2 4BW, UK; ruth.valentine@newcastle.ac.uk (R.A.V.); anne.maguire@newcastle.ac.uk (A.M.); 5 School of Health and Life Sciences, Teesside University, Middlesbrough TS1 3BA, UK

**Keywords:** fluoride, exercise, metabolism, genetic variability

## Abstract

We compared the parameters related to glucose homeostasis, and liver and muscle proteomes in fluorosis-susceptible (A/J; S) and fluorosis-resistant (129P3/J; R) mice in response to fluoride (F) exposure and exercise. Ninety male mice (45 R-mice and 45 S-mice) were randomized into three groups: (SI; RI) No-F, No-Exercise, (SII; RII) 50 ppm F, No-Exercise, (SIII; RIII) 50 ppm F, Exercise. Overall, mean F concentrations in the plasma and femur were significantly higher in R-mice compared with S-mice. In R-mice, exercise resulted in an increase in F accumulation in the femur. In S-mice, the mean plasma glucose level was significantly higher in Group II compared with Groups I and III. There was an increase in liver proteins involved in energy flux and antioxidant enzymes in non-exercise groups (I, II) of S-mice in comparison with the corresponding groups of R-mice. The results also showed a decrease in muscle protein expression in Group I S-mice compared with their R-mice counterparts. In conclusion, the findings suggest an increased state of oxidative stress in fluorosis-susceptible mice that might be exacerbated by the treatment with F. In addition, fluorosis-susceptible mice have plasma glucose levels higher than fluorosis-resistant mice on exposure to F, and this is not affected by exercise.

## 1. Introduction

The importance of fluoride (F) is well recognized by the scientific community. This element plays a key role in achieving and maintaining oral health due to its potential to control the development of dental caries lesions. For this reason, F has been employed in public health programs and included in dental products, such as toothpaste, that have long been recognized as the main reason for the decline of caries in Western countries [1]. However, the “therapeutic window” of F is rather narrow. All sources of F contribute to the total daily F intake. Thus, it is highly desirable to know the appropriate level of exposure of the organism to F in order to minimize the risks and maximize the benefits arising from exposure [2].

Upon excessive ingestion, F interferes with the major metabolic pathways of the body’s biological systems, functioning as a potent inhibitor of many enzymes, including those from the glycolytic pathway [3]. Only a small amount can be tolerated by living cells and excessive F can cause several biochemical changes [4,5]. The effects appear to be dose and time-dependent [3,5,6,7] and influenced by genetics [2,8], which makes it difficult to precisely determine the “optimal” range of F intake that is able to prevent dental caries with minimum side-effects [2]. Mice susceptible (A/J) or resistant (129P3/J) to F have been evaluated to better understand how genetic background impacts the effects of F [9]. It has been shown that 129P3/J mice excrete lower amounts of F in urine, which leads to higher circulating F levels when compared to their A/J counterparts, as well as increased F accumulation in the femur of 129P3/J mice [10]. However, 129P3/J mice are remarkably resistant to the occurrence of dental fluorosis, having a slower onset and less severe development of these lesions [10]. 

Another factor affecting F metabolism is exercise, although the mechanism behind this effect is not precisely known [11]. The pharmacokinetics of F may be altered by changes in physiological responses to physical exercise. When F is used for the prevention of dental caries, the timing of F ingestion may provoke changes in body F retention that could influence the effect of F on tooth and bone development [12]. To date, only three studies evaluated the effects of physical exercise on F metabolism in rodents. In two of them, light exercise decreased plasma F concentrations of rats [13,14]. However, a recent study with A/J mice found no effect of intensive exercise on plasma F levels in A/J mice [15]. It is known that physical exercise provokes changes in gene expression and protein synthesis [16] that may be mediated by the content of exosomes [17], improving glucose homeostasis and vascular protection. On the other hand, the effects of F on glucose homeostasis are not precisely known currently. While some studies have reported beneficial effects of F [18,19,20,21], others have not [13,22,23]. In addition, the effect of F on glucose homeostasis in A/J and 129P3/J mice is not known so far; however, interestingly, an epidemiological study revealed that fluoridation of drinking water was able to predict the prevalence and incidence of age-adjusted Type 2 Diabetes in 22 US states [24].

The present study aims to elucidate the complex interplay between genetics and lifestyle (exercise) factors on the effects of F on glucose homeostasis by assessing alterations in the proteomic profile of the liver and muscles of mice with distinct susceptibilities to the effects of F.

## 2. Results

### 2.1. Body Weight and F Intake

The body weight at the beginning of the study was similar among the groups and increased over the weeks. Two-way ANOVA did not detect a significant difference between the strains or treatments (*p* > 0.05). Table 1 shows descriptive data relative to the mean amount of F ingested from water, diet, as well as total intake (water + diet) during the eight weeks of the study. In relation to the F ingested from water, animals in group SIII ingested less F compared to the other groups. The intake of F from the diet was similar between the strains and groups, while the highest F intake was due to increased F intake from water. 

### 2.2. Exercise Training

Results of the maximum running speed test are presented in Table 2, according to the strain, F exposure and exercise regime. Regardless of the strain, the groups that underwent high-intensity interval training (HIIT) had significant increases in the running speed when compared with the sedentary groups. It should be highlighted that exposure to F did not change the mean running speed only in comparison to the unexposed groups. Regarding the final performance (minutes in the treadmill at the end of the training protocol), according to the last maximum test, for the comparison between the strains, RII mice had values significantly higher when compared with their SII counterparts. For the comparison among the treatments, for both strains, group III had values significantly higher than group II.

### 2.3. Fluoride Analyses in Plasma, Liver and Femur

For plasma F, F-exposed R mice had significantly higher plasma F concentrations than their S counterparts. When the treatments were compared, F-exposed groups had significantly higher plasma F concentrations compared with control, regardless of exercise. Regarding the femur F concentrations, similar to what was observed for plasma, F-exposed R mice had significantly higher femur F concentrations than their S counterparts. For the comparison among the treatments, exposure to F significantly increased bone F concentrations compared with control. Moreover, RIII mice had significantly higher bone F levels than RII ones, but the same was not observed for S mice. For the liver, in the intra-lineage comparison, the only difference observed was for the 129P3/J strains, the RIII group had higher F concentrations in the liver compared to RI and RII. While comparing treatments, regardless of strain, physical training (groups III) led to significantly higher F concentrations in the liver compared to sedentary animals (groups II) (Table 3).

### 2.4. Analysis of Plasma Glucose and Insulin and Calculation of the HOMA2-IR

Regarding glucose homeostasis parameters, the SII had significantly higher plasma glucose levels than their RII counterparts. In line with this, SII mice had a significantly lower % when compared with their RII counterparts. There were no significant differences in the other parameters (Table 3).

**Figure 1:**
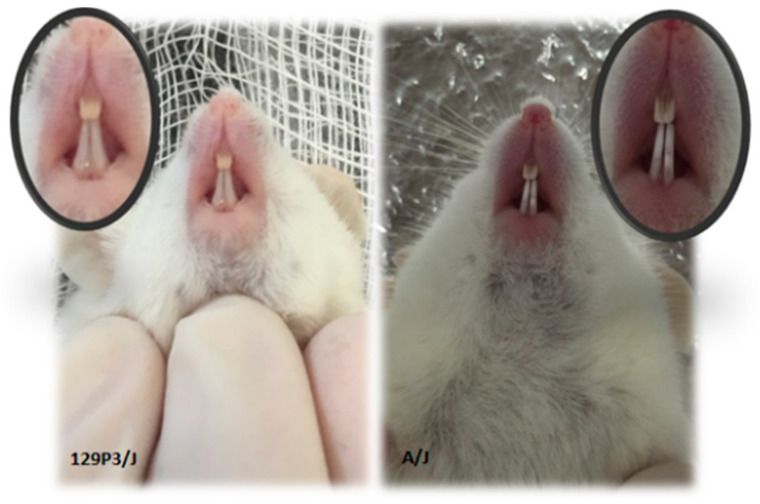
Representative pictures of incisors of susceptible (S; A/J) and resistant (R, 129P3/J) mice that ingested water with 50 ppm of F for wight weeks. Incisors of A/J mice presented a whiter color, indicating greater severity of dental fluorosis.

The examination of the incisors of animals from SII and RII groups (exposed to 50 ppm F) showed the presence of fluorosis in both, but visually, SII animals presented with whiter teeth, indicating a greater severity of dental fluorosis (Figure 1). 

### 2.5. Liver and Gastrocnemius Muscle Proteomic Analysis

For the proteomic analysis in the liver, when the strains were compared, an increase in protein expression was seen for the non-exercised A/J mice; this increase was greater when the animals received deionized water (Table 4).

**Figure 2:**
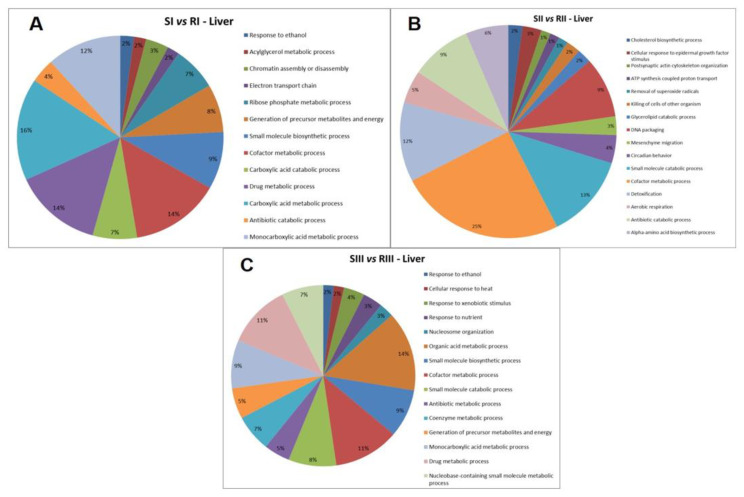
Functional distribution of proteins identified with differential expression in the liver of A/J and 129P3/J mice for each comparison. Comparisons are: (**A**) SI (A/J, deionized water, no-exercise) vs. RI (129P3/J, deionized water, no-exercise); (**B**) SII (A/J, water containing 50 ppm F, no-exercise) vs. RII (129P3/J, water containing 50 ppm F, no-exercise); (**C**) Scheme 50. ppm F, exercise) and RIII (129P3/J, water containing 50 ppm F, exercise). Categories of proteins based on GO annotation Biological Process. Terms significant (Kappa = 0.4) and distribution according to the percentage of a number of gene associations.

**Figure 3:**
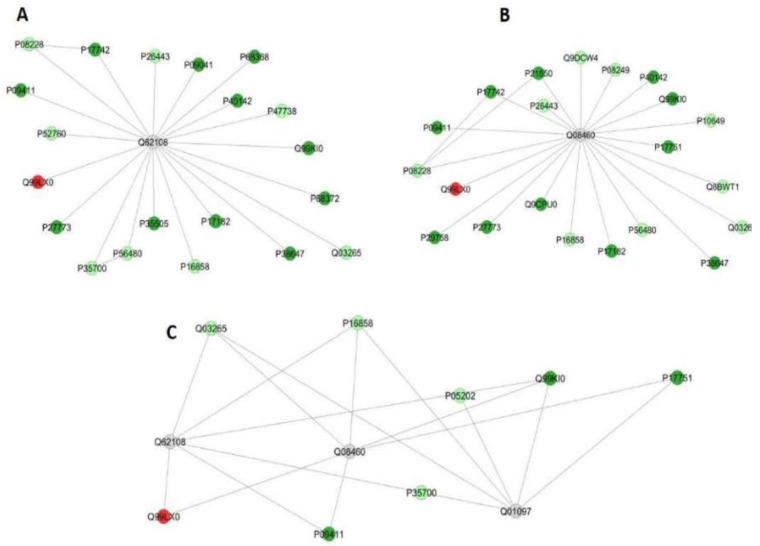
Subnetworks generated by ClusterMarker^®^ for the comparison SI (A/J, deionized water, no-exercise) vs. RI (129P3/J, deionized water, no-exercise) for liver (**A**–**C**). The color of the nodes indicates the differential expression of the respective protein with its access code, available from UniProt protein database (http://www.uniprot.org/, accessed on 20 July 2021). The dark red and dark green nodes indicate proteins unique to RI and SI groups, respectively. The light red and light green nodes indicate down- and upregulated proteins, respectively, in SI group in respect to RI. The gray nodes indicate the interaction proteins that are offered by CYTOSCAPE^®^, which were not identified in the present study.

The functional classification based on GO annotation showed that the category with the highest percentage of the number of gene associations was Carboxylic acid metabolic process (16%) (Figure 2A). The interaction subnetworks (Figure 3) revealed that most of the proteins with changed expression interacted with Disks large homolog 4 (Q62108) and Calcium-activated potassium channel subunit alpha-1 (Q08460). SI mice (A/J) had increased expression or unique proteins mainly related to energy metabolism, involved both in glycolysis (such as Glyceraldehyde-3-phosphate dehydrogenase, Phosphoglycerate kinase, Enolase, Triosephosphate isomerase), Kreb’s cycle (Malate dehydrogenase and dehydratase) and oxidative phosphorylation (ATP synthase and Electron transfer flavoprotein) (Table 5). Isoforms of 3-ketoacyl-CoA thiolase_peroxisomal and of Glutathione S-transferase, as well as Peroxiredoxin-4 (O08807) and Carbonic anhydrase 3 (P16015) were increased more than twofold in the A/J mice (Table S1).

**Figure 4:**
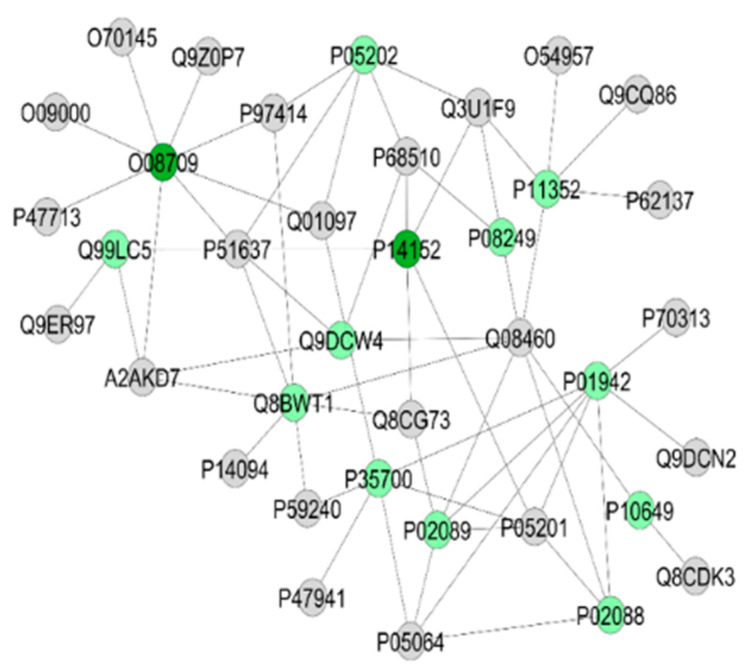
Subnetworks generated by ClusterMarker^®^ for the comparison SII (A/J, water containing 50 ppm F, no-exercise) vs. RII (129P3/J, water containing 50 ppm F, no-exercise) for the liver. The color of the nodes indicates the differential expression of the respective protein with its access code, available from UniProt protein database (http://www.uniprot.org/, accessed on 20 July 2021). The dark green nodes indicate proteins unique to RII and SII groups, respectively. The light green nodes indicate down and upregulated proteins, respectively, in SII group in respect to RII. The gray nodes indicate the interaction proteins that are offered by CYTOSCAPE^®^, which were not identified in the present study.

Upon exercise (SIII vs. RIII), there was a reduction in the total number of proteins found in A/J mice compared to their 129P3/J counterparts (Table 4). The category with the highest percentage of the number of gene associations was Cofactor metabolic process (25%) (Figure 2B). In the interaction subnetwork (Figure 4), proteins with increased expression in SII compared with RII or exclusively found in SII were related to energy flux [(such as Electron transfer flavoprotein subunits alpha and beta, mitochondrial, 3-ketoacyl-CoA thiolase, mitochondrial, Aspartate aminotransferase, mitochondrial, and Malate dehydrogenase (both cytoplasmic and mitochondrial forms)] and oxidative stress (Peroxiredoxin-1 and -6, Glutathione S-transferase Mu 1 and Glutathione Peroxidase 1) (Table 5), as seen for the comparison SI vs. RI.

**Figure 5:**
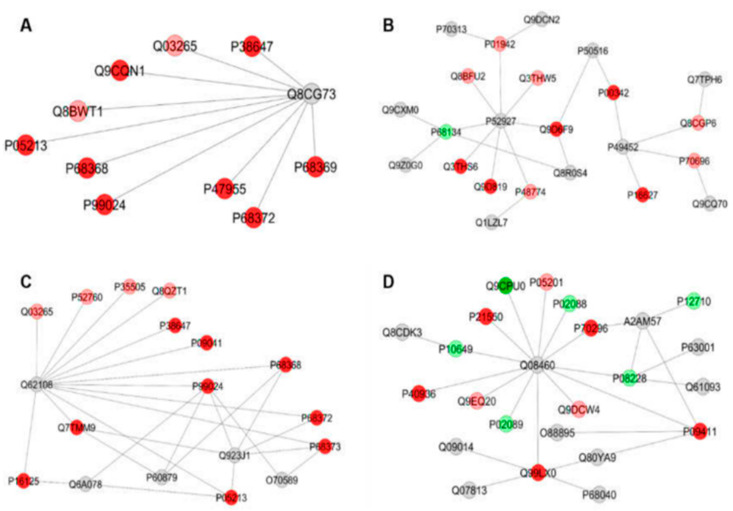
Subnetworks generated by ClusterMarker^®^ for the comparison SIII (A/J, water containing 50 ppm F, exercise) vs. RIII (129P3/J, water containing 50 ppm F, exercise) for liver (**A**–**D**). The color of the nodes indicates the differential expression of the respective protein with its access code, available from UniProt protein database (http://www.uniprot.org/, accessed on 20 July 2021). The dark red and dark green nodes indicate proteins unique to RIII and SIII groups, respectively. The light red and light green nodes indicate down and upregulated proteins, respectively, in SIII group in respect to RIII. The gray nodes indicate the interaction proteins that are offered by CYTOSCAPE^®^, which were not identified in the present study.

Upon exercise, there was a reduction in protein expression in the A/J mice, compared with their 129P3/J counterparts (Table 4). The category with the highest percentage of gene associations was Organic acid metabolic process (14%) (Figure 2C). In the interaction subnetworks (Figure 5A–D), most of the proteins with altered expression in SIII compared with RIII were related to energy flux (Table 5) and interacted with Protein fantom (Figure 5A), High mobility group protein HMGI-C (Figure 5B), Disks large homolog 4 (Figure 5C) and Calcium-activated potassium channel subunit alpha-1 (Figure 5D). The latter two were also interacting partners in the comparison between groups SI vs. RI. Several pathways of the energy metabolism were impaired in SIII when compared with RIII. Important enzymes of aerobic (Phosphoglycerate kinases 1 and 2, as well as beta enolase) and anaerobic (L-lactate dehydrogenase B and C chains) glycolysis were absent in SIII group (Table 5). Also, enzymes involved in amino acid metabolism were reduced (Table 5), such as Fumarylacetoacetase (more than twofold; Table S3). Enzymes involved in oxidative phosphorylation, such as electron transfer flavoprotein subunit beta and ATP synthase subunit alpha, mitochondrial, were reduced in the SIII group (Table 5). Chaperones, such as Heat shock protein 75 kDa, mitochondrial, were absent in SIII. There was the absence of Protein/nucleic acid deglycase DJ-1 in the SIII group. Indolethylamine N-methyltransferase, was also absent in the SIII group, while 2-iminobutanoate/2-iminopropanoate deaminase was reduced.

In the gastrocnemius muscle (Table 6), untreated A/J mice showed a decrease in protein expression when compared with their 129P3/J counterparts (comparison SI vs. RI).

**Figure 6:**
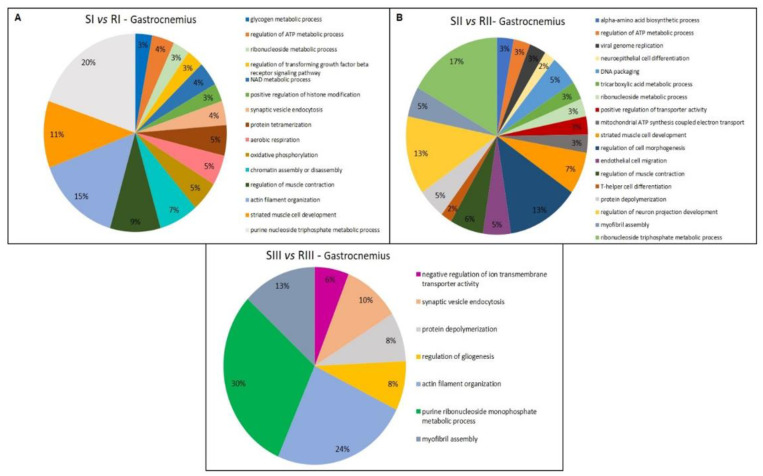
Functional distribution of proteins identified with differential expression in the gastrocnemius muscle of A/J and 129P3/J mice for each comparison. Comparisons are: (**A**) SI (A/J, deionized water, no-exercise) vs. RI (129P3/J, deionized water, no-exercise); (**B**) SII (A/J, water containing 50 ppm F, no-exercise) vs. RII (129P3/J, water containing 50 ppm F, no-exercise); (**C**) SIII (A/J, water containing 50 ppm F, exercise) and RIII (129P3/J, water containing 50 ppm F, exercise).

**Figure 7:**
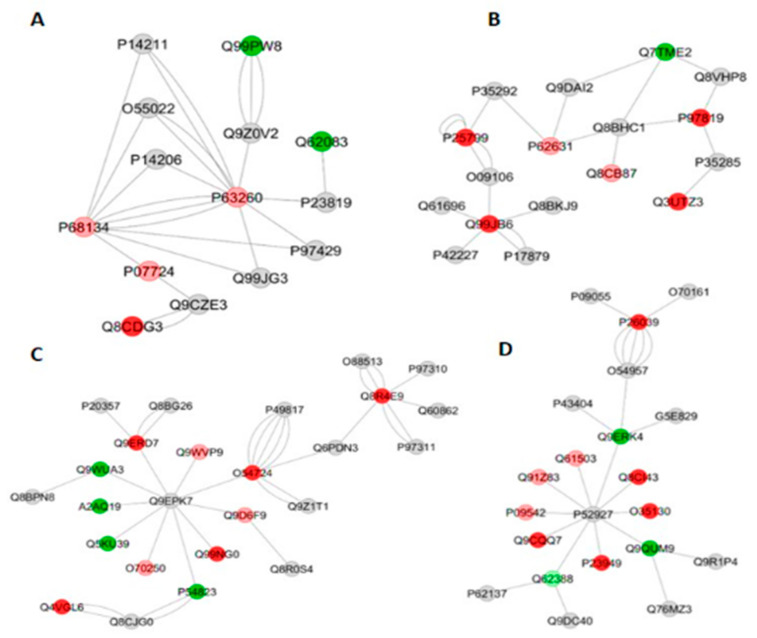
Subnetworks generated by ClusterMarker^®^ for the comparison SI (A/J, deionized water, no-exercise) vs. RI (129P3/J, deionized water, no-exercise) for gastrocnemius muscle (**A**–**D**). The color of the nodes indicates the differential expression of the respective protein with its access code, available from UniProt protein database (http://www.uniprot.org/, accessed on 20 July 2021). The dark red and dark green nodes indicate proteins unique to RI and SI groups, respectively. The light red and light green nodes indicate down and upregulated proteins, respectively, in SI group in respect to RI. The gray nodes indicate the interaction proteins that are offered by CYTOSCAPE^®^, which were not identified in the present study.

The functional classification based on GO annotation showed that the category with the highest percentage of number of gene associations was Purine nucleoside triphosphate metabolic process (20%) (Figure 6A). The interaction subnetworks showed several proteins involved in muscle contraction downregulated or absent in the SI group, (Table 7), such as Actin alpha, Skeletal muscle (P68134), Actin, cytoplasmic 2 (P63260) (Figure 7A), Myosin light chain 6B (Q8CI43), Myosin 7 (Q91Z83) and Myosin light chain 3 (P09542) (Figure 7D). Parvalbumin alpha (P32848), Glyceraldehyde-3-phosphate dehydrogenase (P16858) and Filamin-C (Q8VHX6) were decreased more than 2-fold in the A/J mice (Table S4). Many altered proteins (mostly downregulated or absent in SI mice) are related to protein synthesis (Table 7), such as Helicase ARIP4 (Q99NG0), Roquin (Q4VGL6) (Figure 7C), a post-translational repressor of mRNA, Nuclear factor NF-kappa-B p105 subunit (P25799) (Figure 7B), a transcription factor, mRNA decay activator protein ZFP36L2 (P23949) that promotes poly(A) tail removal or deadenylation of mRNA thus attenuating protein synthesis (Figure 7D), Forkhead box protein P3 (Q00JB6), a transcriptional regulator and Elongation factor 1-alpha 2 (P62631) (Figure 7B). 

Categories of proteins based on GO annotation Biological Process. Terms significant (Kappa = 0.4) and distribution according to the percentage of number of gene associations.

**Figure 8:**
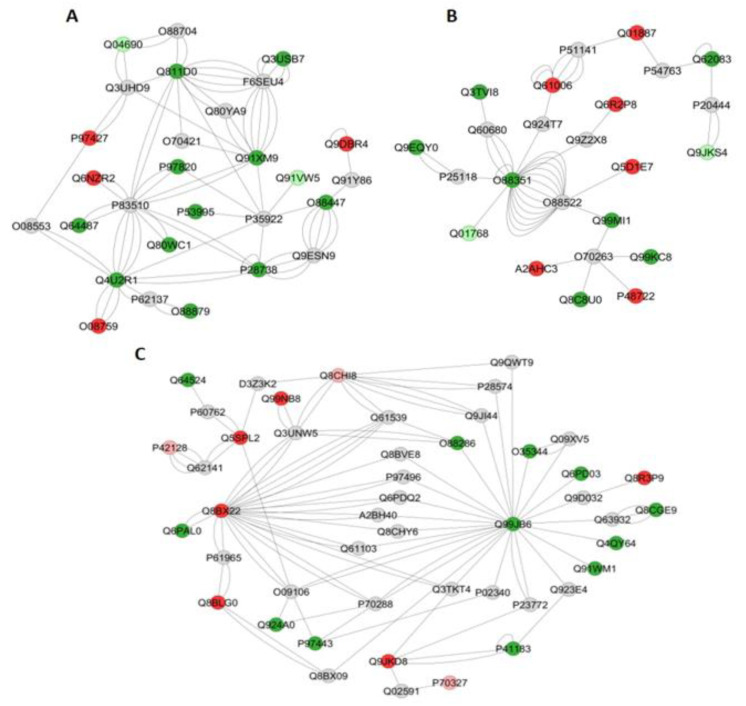
Subnetworks generated by ClusterMarker^®^ for the comparison SII (A/J, water containing 50 ppm F, no-exercise) vs. RII (129P3/J, water containing 50 ppm F, no-exercise) for gastrocnemius muscle (**A**–**C**). The color of the nodes indicates the differential expression of the respective protein with its access code, available from UniProt protein database (http://www.uniprot.org/, accessed on 20 July 2021). The dark red and dark green nodes indicate proteins unique to RII and SII groups, respectively. The light red and light green nodes indicate down and upregulated proteins, respectively, in SII group in respect to RII. The gray nodes indicate the interaction proteins that are offered by CYTOSCAPE^®^, which were not identified in the present study.

Upon exposure to F, associated or not with exercise, an increase in protein expression was seen for the A/J mice compared with the respective 129P3/J mice groups (Table 6). For the sedentary groups that consumed F (SII vs. RII comparison), the functional classification based on GO annotation showed that the category with the highest percentage of number of gene associations was Ribonucleoside triphosphate metabolic process (17%) (Figure 6B). In the interaction subnetworks, several proteins with increased expression or exclusively found in SII group interacted with Traf2 and NCK-interacting protein kinase (P83510) (Table 7). Among the interacting partners were Anaphase-promoting complex subunit 1 (P53995), related to ubiquitination, Mitogen-activated protein kinase kinase kinase kinase 4 (P97820), Disks large homolog 1 (Q811D0) and Kinesin light chain 1 (O88447) (Figure 8A). Interestingly, exposure to F increased more than 2-fold proteins related to muscle contraction and relaxation, as well as proteins related to energy flux (Table 7). 

Among the interacting partners were Anaphase-promoting complex subunit 1 (P53995), related to ubiquitination, Mitogen-activated protein kinase kinase kinase kinase 4 P97820), a serine/threonine kinase that plays a role in response to environmental stress, Disks large homolog 1 (Q811D0) that acts in signal transduction and Kinesin light chain 1 (O88447) that is a microtubule-associated force-producing protein playing a role in organelle transport (Figure 8A). In addition, some proteins with altered expression interacted with players involved in the regulation of nuclear factor kappa-B (NF-kB), as well as Inhibitor of nuclear factor kappa-B kinase subunit alpha (Q60680), NF-kappa-B essential modulator (O88522) and E3 ubiquitin-protein ligase RNF31 (Q924T7). Among the proteins interacting with them were Inhibitor of nuclear factor kappa-B kinase subunit beta (O88351 that was exclusively found in the SII group, and Endoribonuclease ZC3H12A (Q5D1E7) (Figure 8B) that was exclusively identified in the 129P3/J animals. Proteins related to muscle contraction and relaxation were increased more than 2-fold in the SII mice (Table 5), such as Parvalbumin alpha (P32848; more than 18-fold increase), Tropomyosin alpha-3 chain (P21107; more than 3-fold increase), Myosin-7B (A2AQP0) and Troponin I, fast skeletal muscle (P13412) and Calsequestrin-1 (O09165) (Table S5). Proteins related to energy flux were also increased more than 2-fold in the SII mice (Table 5), such as Glyceraldehyde-3-phosphate dehydrogenase testis-specific (Q64467; more than 6-fold), Fatty acid-binding protein, heart (P11404; more than 3-fold), AMP deaminase 1 (Q3V1D3) and ADP/ATP translocases (isoforms 1 and 2) (Table S5).

**Figure 9:**
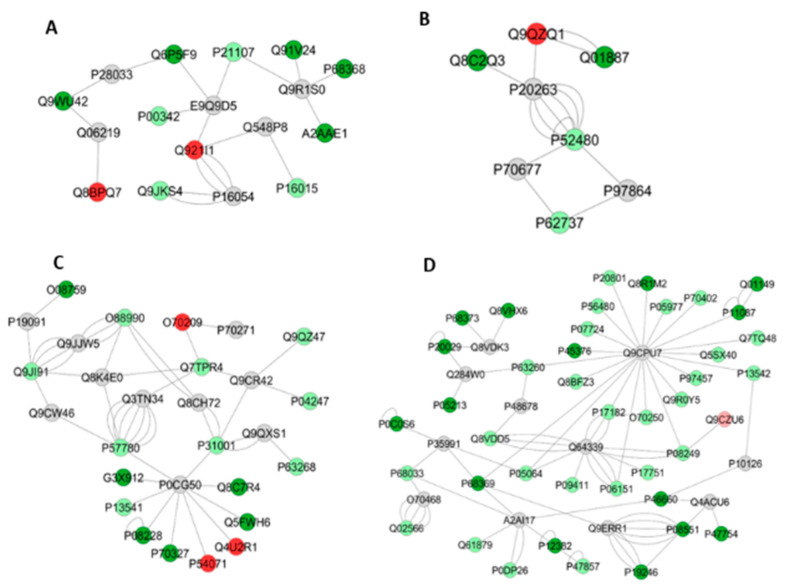
Subnetworks generated by ClusterMarker^®^ for the comparison SIII (A/J, water containing 50 ppm F, exercise) vs. RIII (129P3/J, water containing 50 ppm F, exercise) for gastrocnemius muscle (**A**–**D**). The color of the nodes indicates the differential expression of the respective protein with its access code, available from UniProt protein database (http://www.uniprot.org/, accessed on 20 July 2021). The dark red and dark green nodes indicate proteins unique to RIII and SIII groups, respectively. The light red and light green nodes indicate down and upregulated proteins, respectively, in SIII group in respect to RIII. The gray nodes indicate the interaction proteins that are offered by CYTOSCAPE^®^, which were not identified in the present study.

For the exercised mice (SIII vs. RIII comparison), based on GO annotation the category with the highest percentage of number of gene associations was Purine ribonucleoside monophosphate metabolic process (30%; Figure 6C). The interaction subnetworks (Figure 9), as well as Table S6 showed that for the SIII group, there was an increase or exclusivity in the expression of proteins related to energy flux (Krebs cycle and glycolytic pathway) (Table 7), such as Malate dehydrogenase (P08249), Glyceraldehyde-3-phosphate dehydrogenase (Q64467), L-lactate dehydrogenase chain (P00342), Triosephosphate isomerase (P17751), Phosphoglycerate kinase (P09411), Pyruvate kinase PKM (P52480) and Alpha-(P17182), Beta- (P21550) and Gamma- (P17183) enolase. Similar to what was reported for SII mice, proteins related to muscle contraction/relaxation considerably increased in SIII mice (Table 5 and Table S6).

## 3. Discussion

Many factors have been shown to affect the metabolism of F, with the potential to interfere in the retention of this ion in the organism and alter the relationship between F intake and the risk of fluorosis, as well as glucose homeostasis [2,11]. Among these factors are the genetic background that has been extensively studied using A/J and 129P3/J mice [7,10,25,26,27], as well as lifestyle factors, such as physical exercise [12,13,14,15]. In the above-mentioned studies, the effects of genetic and lifestyle factors on F metabolism and/or glucose homeostasis were studied separately. This is the first study to evaluate the interplay between genetic and lifestyle factors on F metabolism and glucose homeostasis. We studied mice with different susceptibilities to the effects of F in the organism [8,9,10] that were exposed to F only or to both F and physical exercise. R mice treated with F, regardless of exercise, had plasma F levels significantly higher than their S counterparts (Table 3). Despite the higher plasma F levels, the first had lower severity of dental fluorosis, in line with previous studies [10,26]. However, high F levels that were administered to the animals appeared not to have induced toxicity since their body weight did not change when compared with the animals that were not treated. 

Regarding the protocol of exercise, the R animals had a better performance at the final test in comparison with their S counterparts (Table 2). This was somehow expected since the S animals are more susceptible to the toxic effects of F in the organism [8,9,10]. F has a great affinity for hydroxyapatite and becomes rapidly taken up by bone, which results in 99% of the F body burden being associated with calcified tissues [11]. R mice exposed to F have been reported to have higher plasma and femur F concentrations compared with their S counterparts [10], which was confirmed in the present study. We observed that physical exercise did not impact plasma F concentrations (Table 3) but led to significantly higher femur F concentrations for the R mice only, indicating higher retention of F in the hard tissues of these mice. This increase in femur F concentrations upon exercise had been previously reported in Sprague Dawley rats and is associated with lower F toxicity since F incorporation in bone reduces plasma F levels [13]. It should be noted that trained S mice did not present a significant increase in femur concentrations compared to their counterparts that were only exposed to F, which is in line with our previous study [15] but is different from what was reported by Lombarte et al. [13] for Sprague Dawley rats. These results indicate a complex interplay between genetics and exercise in F metabolism, which might impact the relationship between the amount of F intake and the risk of developing dental fluorosis. In general, according to the protocol of the present study, plasma F levels were not affected by physical exercise, regardless of the genetic background. However, exercise increased F taken up by bone in R mice. It is important to highlight that bone is a F reservoir since F can be released back to the systemic circulation upon bone resorption [11] and this could impact dental fluorosis, which should be evaluated in further studies.

After the determination of the appropriate training protocol, the study commenced and the animals submitted to the developed HIIT protocol [15]. The genetic factors represent a substantial portion of resistance to physical training, with a heritability estimated at around 50% [28]. When different mice strains were analyzed, it was observed that genetic variability substantially influenced the resistance capacity; this influence is evident even when different types of motor activities are explored [29,30,31]. The genetic background of R and S strains did not affect neither their final physical capacity or their change in maximum running speed during the experimental period (Table 2). Upon exposure to F, genetics influenced the final physical capacity since the S sedentary mice had this parameter significantly lower than their R counterparts, but this deleterious effect was counteracted by physical exercise. Therefore, a sedentary condition creates greater risks to physical capacity after exposure to F.

Regarding the parameters related to glucose homeostasis, the only differences found were for plasma glucose and %B for the different strains. RII mice had plasma glucose levels significantly lower and %B significantly higher than their SII counterparts (Table 3). These data indicate that glucose homeostasis might be more influenced by genetic than by lifestyle factors (exercise). This might help to explain the conflicting results reported in previous studies. Wistar rats with streptozotocin-induced diabetes had increased insulin sensitivity when exposed to water containing 10 ppm F [19], while Sprague Dawley rats exposed to water containing 15 ppm F had increased insulin resistance [13]. Moreover, non-obese diabetic (NOD) mice exposed to water containing 10 ppm F had reduced plasma glucose levels when compared to their non-exposed counterparts [20,21]. 

Regarding the proteomic analysis of the liver, S non-exercised mice, especially when drinking deionized water, had an increase in protein expression (Table 4). This is in accordance with a recent study by our group when the liver proteome of these two mice strains was compared and might be explained by an increase in Formimidoyltransferase-cyclodeaminase (Q91XD4; Table S1) [32] that was exclusively identified in the S mice in the present study. This enzyme is involved in the synthesis of purines and pyrimidines, as well as amino acids [33]. It should be noted that the category with the highest percentage of a number of gene associations was the Carboxylic acid metabolic process, and the proteins with increased expression and also uniquely expressed in S mice were related to different pathways of energy metabolism, such as glycolysis, Kreb’s cycle and oxidative phosphorylation (Table 5 and Table S1). Increased energy flux leads to oxidative stress, which agrees with an increase in antioxidant enzymes in S mice not exposed to F compared with their R counterparts. These findings were also observed in a previous study of our group [32]. The increase in proteins related to energy flux and antioxidant proteins in S mice might be a plausible explanation for their high susceptibility to the effects of F, since this ion is well known for its ability to induce oxidative stress [3,4,32].

When exposed to F, S mice still had increased protein expression (mainly in proteins involved in energy flux and oxidative stress) in the liver, although to a lower extent when compared with their R counterparts. Interestingly, the category with the highest percentage of a number of gene associations was Cofactor metabolic process (Figure 2B). This might be associated with the fact that fluorine is the most electronegative element in the periodic table and has a high affinity for metal ions [11], which act as cofactors for several enzymes. It should be highlighted that Fructose-1,6-bisphosphatase 1 (Q9QXD6), which acts as a rate-limiting enzyme in gluconeogenesis, was increased more than 2-fold in the SII group (Table S2). Fluoride is known as a potent inhibitor of glycolytic enzymes, such as enolase, hexokinase, phosphofructokinase, and pyruvate kinase, due to its strong ability to bind metals [34]. Thus, impairment in glycolysis by F might activate the metabolism of other fuels, which is in line with the increase in Aspartate aminotransferase_mitochondrial, 3-ketoacyl-CoA thiolase, mitochondrial, and Fructose-1,6-bisphosphatase 1.

When the mice were exposed both to F and exercise, the profile of protein expression changed remarkably, i.e., SIII mice had a reduction in liver proteins expression when compared with RIII. Several proteins with reduced expression are involved in distinct pathways of energy metabolism, such as aerobic and anaerobic glycolysis, oxidative phosphorylation, and amino acids metabolism. Important detoxifying proteins were absent in SIII mice, such as protein/nucleic acid deglycase DJ-1 that prevents the formation of advanced glycation products (AGEs) [33]. The presence of High mobility group protein HMGI-C (Figure 3B), a transcription regulator, among the interacting proteins, in addition to the absence of 60S acidic ribosomal protein P1, is essential to the elongation step of protein synthesis and the absence of S-adenosylmethionine synthase isoform type-2, involved in the regulation of protein expression, as well as the reduction in more than 2-fold of in Elongation factor 1-alpha 2 (P62631; Table S3) might help to explain the reduced protein synthesis in SIII mice compared with RIII. Additionally, the quality control of synthesized proteins might have been impaired due to the absence of Heat shock 70 kDa protein 1-like, an essential molecular chaperone implicated in a wide variety of cellular processes [33]. In summary, when the liver proteome of the S and R strains is compared, in the presence of no stressor or one stressor (F only), there is an increase in protein synthesis, energy flux, and antioxidant enzymes in the first (SI compared to RI and SII compared to RII). However, in the presence of two stressors (F and exercise), there is a remarkable reduction in proteins involved in protein synthesis, energy metabolism, and detoxification, but antioxidant enzymes are still increased in the S mice (SIII compared to RIII mice). These results, when analyzed in conjunction with the literature, suggest an increased state of oxidative stress in S mice that is inherent to this strain and might be exacerbated by the treatment with F, which is also known to provoke oxidative stress [3,7,32].

Regarding the gastrocnemius muscle (Table 7), separately from which was observed for the liver (Table 5), untreated S mice showed a decrease in protein expression when compared with their R counterparts (comparison SI vs. RI), which might be due to downregulation or absence of several proteins involved in protein synthesis (Table 7). Remarkably, the SI group had downregulation or absence of several proteins related to muscle contraction. Proteins such as Parvalbumin alpha (P32848), involved in relaxation after contraction, as well as Glyceraldehyde-3-phosphate dehydrogenase (P16858) and Filamin-C (Q8VHX6) that function as large actin-cross-linking protein critical for maintaining the structural integrity of the muscle fibers [33], were decreased more than 2-fold in the A/J mice (Table S4). This might indicate impaired muscle contraction in the S mice, even in the absence of stressors. However, the maximum velocity test was reduced in the S mice compared with the R ones only in the F-treated groups (SII vs. RII). Exposure to F, associated with or not to exercise, provoked an increase in protein expression in the S mice compared to their respective R counterparts, similarly to what was found for the liver (Table 4). In the interaction subnetworks, some proteins with altered expression interacted with proteins involved in the regulation of nuclear factor kappa-B (NF-kB). Among them is an inhibitor of nuclear factor kappa-B kinase subunit beta (O88351), a serine kinase playing an essential role in the activation of the (NF-kB) signaling pathway by cellular stresses that were exclusively found in the SII group, as well as Endoribonuclease ZC3H12A (Q5D1E7) (Figure 8B). This enzyme, exclusively identified in the R mice, is an endoribonuclease that prevents NF-kB signaling pathway activation, negatively regulating macrophage-mediated inflammatory response and immune homeostasis [33]. NF-kB, as a transcription factor, plays a crucial role in immune and inflammatory responses via the regulation of genes expression. 

In a non-stimulated state, NF-kB exists mainly in the cytoplasm, combined with the inhibitory protein B (IkBs). When activated by some stimulators, including pro-inflammatory cytokines, bacteria, lipopolysaccharide (LPS), viruses, physical or chemical stresses, the IkB proteins become phosphorylated and disconnect with NF-kB, which triggers NF-kB translocation to the molecule and binding to their cognate DNA binding sites to regulate the transcription of its downstream genes. Some of the genes are related to inflammatory responses, such as pro-inflammatory cytokines, chemokines, adhesion molecules, and inducible enzymes such as cyclooxygenase-2 (COX2) and iNOS [35]. Several studies have reported that treatment with F increases NF-kB in distinct cells, such as neurons [36], renal and cardiac cells [37]. A recent study showed that treadmill running reduced excessive activation of microglia in the hippocampus of mice treated with 24 mgF/kg, together with changes in the pathway of neuroactive ligand-receptor interaction [38]. Our results showed increased expression of proteins related to NF-kB pathway activation in the muscle of susceptible animals treated with F (SII), while the resistant animals (RII) had an increase in proteins that prevent NF-kB signaling pathway activation. This might be another probable mechanism that helps to explain the resistance of the 129P3/J mice to the effects of F. 

Proteins related to muscle contraction and relaxation, such as Parvalbumin alpha (P32848), Tropomyosin alpha-3 chain (P21107), Myosin-7B (A2AQP0) and Troponin I, fast skeletal muscle (P13412), and Calsequestrin-1 (O09165) (Table S5), as well as proteins related to energy flux, such as Glyceraldehyde-3-phosphate dehydrogenase testis-specific (Q64467), Fatty acid-binding protein, heart (P11404), AMP deaminase 1 (Q3V1D3) and ADP/ATP translocases (isoforms 1 and 2) (Table 7), were increased more than 2-fold in the SII mice. Curiously, some of these proteins, such as Parvalbumin alpha and Glyceraldehyde-3-phosphate dehydrogenase were reduced more than 2-fold in the untreated S mice (comparison SI vs. RI; Table S4). Moreover, an increase in Parvalbumin alpha was also recently described in the muscle of NOD mice treated with 50 ppm fluoride [20]. In addition, Neurofibromin (Q04690), which stimulates the GTPase activity of Ras, regulating its activity, was also increased more than 2-fold in the SII mice (Table S5), indicating the involvement of MAPK/ERK signaling pathways in the muscle events. It is intriguing why proteins related to contractile function and energy metabolism were increased in SII mice compared with RII counterparts, considering that the former had final performance in the last maximum test significantly lower than the latter, which is in line with our previous study showing a reduction in exercise capacity upon F exposure in A/J mice [15]. Future mechanistic studies should be developed to shed light on this aspect.

Simultaneous exposure to F and exercise caused an increased expression of proteins related to energy flux in SIII mice compared with their RIII counterparts. It was also observed an increase in carbonic anhydrase 3 (P16015) that was also increased in the liver. In fact, this enzyme was consistently increased more than 2-fold in the S mice in comparison with 129P3/J counterparts, regardless of the treatment both in the liver (Tables S1–S3) and gastrocnemius muscle (Tables S4–S6). This enzyme catalyzes the interconversion of carbon dioxide (CO_2_) and water into carbonic acid, protons (H^+^), and bicarbonate (HCO^3−^), playing a crucial role in the acid-basic homeostasis of the organism. Increased carbonic anhydrase (CA) synthesis may be directly induced by a lower oxygen tension at the molecular level [39]. Acid-basic disturbances may alter the metabolism of F in several ways. They can reduce urinary pH, thus increasing F retention in the organism [11]. However, A/J mice, despite being more susceptible to the development of dental fluorosis, were intriguingly shown to have lower circulating F levels than their 129P3/J counterparts [10]. In addition, CA is essential to maintain pH homeostasis in enamel during the maturation stage, when the growth of enamel crystals results in excessive amounts of H+ ions. In this situation, CA is required to avoid the pH of the developing enamel becoming too acidic [40]. In fact, isoforms of CA were identified exclusively in the enamel of A/J mice treated with 50 ppm fluoride, but not in 129P3/J mice [26], which is consistent with the increased levels of this enzyme found in the liver and muscle of A/J mice in the present study. The implications of this in the differential development of dental fluorosis in these two mice strains must be evaluated in further studies. 

## 4. Materials and Methods

### 4.1. Animals, Treatment and Samples Collection

**Figure 10:**
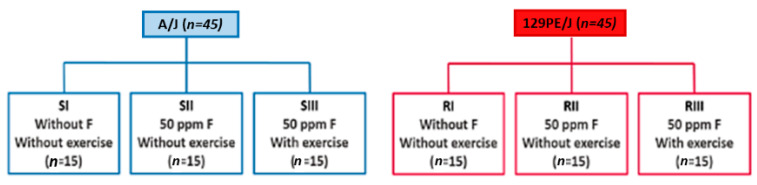
Division of the experimental groups according to the strains (A/J—susceptible (S) to dental fluorosis; 129P3/J—resistant (R) to dental fluorosis) and treatments [I—water without fluoride, no exercise; II—water with 50 ppm fluoride, no exercise; III—water with 50 ppm fluoride, exercise (high-intensity interval training (HIIT) protocol on a treadmill, five days per week for eight weeks)].

This project was approved by the Animal Research Ethics Committee of Bauru School of Dentistry, University of São Paulo (FOB-USP, Proc.009/2015). The sample consisted of ninety 21-day-old male mice, comprising 45 129P3/J strain and 45 A/J strain. The A/J strain (S) is highly susceptible to dental fluorosis, presenting a rapid and severe development of the disease when the animal is exposed to F, while the 129P3/J strain ® is less affected, with low severity of dental fluorosis (Everett et al., 2002). All mice remained in metabolic cages (*n* = 2 per cage), in rooms with temperature, humidity, and controlled light/dark cycles (~23 ± 1 °C, 40–80%, and 12/12 h, respectively). The animals had free access to a diet with a low concentration of F (Presence, Purina, <1 ppm). Animals from both strains (S and R), at 45 days of age, were randomly divided into groups [9] according to their exposure to F (0 or 50 ppm) through the drinking water and physical exercise (daily runs on a treadmill five days per week for 60 min at a high intensity (80% of maximum running speed); III) (Figure 10). The concentration of 50 ppm (as NaF) was chosen to simulate the ingestion of fluoridated water in endemic areas by humans, taking into account that rodents metabolize F 5–10 times faster than humans [41].

Body weight, water and diet were measured once a week. For the experimental groups SIII and RIII, physical exercise was performed five days per week on an appropriate treadmill for 8 weeks [42]. At the end of the study (eight weeks), the animals were fasted for approximately 10 h and then anaesthetized with sodium thiopental. Blood, liver, and femur were collected for F analyses. In addition, blood was also collected and stored at −20 °C for analysis of glucose and insulin. Liver and whole gastrocnemius muscle were collected and stored at −80 °C for proteomic analysis.

### 4.2. Exercise Protocol and Treadmill Training Experiment

All mice were familiarized with the treadmill for 10 min/session at a speed of 8 m/min once a day for 1 week [43]. After the period of familiarization, the mice performed a Maximum Test to determine the Maximum Velocity reached in the treadmill [44]. The test started at a speed of 6 m/min and was increased by 3 m/min every 3 min until exhaustion when the animals stopped running. This Maximal Test was repeated after 4 weeks (to adjust exercise intensity) and at the end of 8 weeks.

The animals of groups SIII and RIII underwent a high-intensity interval training protocol (HIIT), five days per week for eight weeks. The HIIT sessions were adapted [42] and consisted of 5 min of warm-up at 40% of maximum speed and a sequence of short periods (1 min) of intense exercise at 80% of maximum running speed, followed by the recovery time of 3 min. The training sessions ended when the mice completed the distance of 1000 m. All training sessions were held at the same time each day to avoid effects on training performance [45].

### 4.3. Fluoride Analyses in Plasma, Liver and Femur

F concentrations were analyzed in duplicate, as previously described [46]. Analyses were performed with an ion-specific electrode (Orion Research, Model 9409, Massachusetts, MA, USA) and a miniature calomel electrode (Accumet, #13-620-79, Massachusetts, MA, USA), both coupled to a potentiometer (Orion Research, Model EA 940), after hexamethyldisiloxane-facilitated diffusion [14,47]. F standards (0.005–0.950 µg F) were prepared in triplicate and diffused in the same manner as the samples.

### 4.4. Analysis of Plasma Glucose and Insulin and Calculation of the HOMA2-IR

Glycemia was analyzed by the glucose oxidase method, using a commercial kit (Katal Biotecnologia, São Paulo, Brazil). Insulinemia was evaluated by ELISA (Mouse Insulin kit, #80-INSMN-E01, ALPCO Diagnostics, Salem, MA, USA). Both analyses were performed in duplicate, according to the manufacturer’s instructions. HOMA2-IR (homeostasis model assessment 2 of insulin resistance) was evaluated from paired plasma glucose and insulin concentrations, using the software HOMA Calculator v.2.2 (available from http://www.dtu.ox.ac.uk/homacalculator/download.php, accessed on 20 July 2021). The software provides calculation of HOMA2-IR index, as well as insulin sensitivity (% S) and β-cell function (%B) [48].

### 4.5. Liver and Gastrocnemius Muscle Preparation for Proteomic Analysis

For protein extraction from whole gastrocnemius muscle and liver, 50 mg of each tissue from each animal were transferred to a microtube and 500 µL of extraction buffer containing 7 M urea, 2 M thiourea and 40 mM Dithiothreitol (DTT) were added and mechanical homogenization (cryogenic mill) was carried out. After 1-h incubation on ice, with vortexing performed every 10 min, the homogenate was centrifuged at 20,817× *g* for 30 min at 4 °C and the supernatant was collected. After extraction, the proteins levels were quantified by the Bradford method [49]. For each group, twenty-five μg of liver protein from two animals were pooled, in order to obtain biological triplicates. Then, protein samples (50 µg) were transferred to a microtube and 10 µL of 50 mM ammonium bicarbonate and 25 µL of 0.2% RapiGest™ (Waters Co., Manchester, UK) were added and incubated at 37 °C for 60 min. Then they were reduced and alkylated, respectively by incubating with 5 mM DTT (dithiotreitol) at 37 °C for 40 min and 10 mM IAA (iodocetamide) for 30 min in the dark at room temperature. Proteolytic digestion was performed by the addition of 10 µL trypsin (100 ng; Trypsin Gold Mass Spectrometry, Promega, Madison, WI, USA) by incubation for 14 h at 37 °C. After digestion, 10 µL of 5% TFA (trifluoroacetic acid) was added, incubated for 90 min at 37 °C, and then centrifuged (20,817× *g* for 30 min). The supernatant was collected and 5 µL ADH (1 pmol/µL) and 85 µL 3% ACN (acetonitrile) were added so that the samples were destined for nLC-ESI-MS/MS analysis.

### 4.6. nLC-ESI-MS/MS and Bioinformatics Analyses

Identification of the peptides was performed in the nanoAcquity UPLCXevo QTof MS system (Waters, Manchester, UK), as previously described [18]. Differences in protein expression between the groups were obtained by the *t* test, embedded in the Protein Lynx Global Service (PLGS) software version 3.03, (Monte Carlo algorithm) and expressed as *p* < 0.05 for down-regulated proteins and 1 − *p* > 0.95 for upregulated proteins. Comparisons were made between the strains, for each treatment (SI vs. RI, SII vs. RII and SIII vs. RIII).

To understand the biological significance of the quantitative results of the proteomic analysis, the differentially altered proteins in each comparison were analyzed using bioinformatics tools, as previously reported [18,50,51,52]. The software CYTOSCAPE^®^ 3.0.4 (Java^®^) was used to build networks of molecular interaction between the identified proteins, with the aid of ClueGo and ClusterMark applications [53].

### 4.7. Statistical Analysis

Data were analyzed using GraphPad InStat version 3.0 for Windows and GraphPad Prism version 5.0 for Windows software (GraphPad Software Inc., La Jolla, CA, USA), using two-way ANOVA and Sidak’s, Tukey’s or Bonferroni’s tests for *post hoc* comparisons. The criteria evaluated were strain and treatment. In all cases, the level of significance was set at 5%.

## 5. Conclusions

In conclusion, our results suggest an increased state of oxidative stress in S mice that is inherent to this strain and might be exacerbated by the treatment with F. In addition, S individuals might benefit more from the effect of physical exercise on glucose homeostasis than the R ones, upon exposure to F. Our results indicate a complex interplay between genetics and exercise on F metabolism. Exercise seems to increase F accumulation in the bones of R mice, while a sedentary condition reduces the physical capacity in S mice exposed to F. Ultimately, our data indicate that the S mice might benefit more from physical training than the R mice. Studies with similar designs should be conducted with humans presenting different susceptibilities to the effects of F to see if they respond similarly to the interplay between genetics and physical exercise on F metabolism. This topic is of special importance when F is used for the prevention of dental caries, since the timing of F ingestion may affect body F retention and, consequently, the effect of F on tooth and bone development.

## Figures and Tables

**Table 1:** Mean (± SD) F ingested daily during the eight weeks by A/J (susceptible to fluorosis; S) and 129P3/J (resistant to fluorosis; R) mice treated with water containing 50 ppm F or not (control; group I) and submitted to physical exercise (trained; group III) or not (sedentary; group II).

F Intake *		Treatment	
	Control (I)	Sedentary + F (II)	Trained + F (III)
Water (µg)			
A/J (S)	0.00 ± 0.0	1188.9 ± 153.8	858.9 ± 70.8
129P3/J (R)	0.00 ± 0.0	1150.4 ± 139.3	1186.9 ± 167.4
Diet (µg)			
A/J (S)	42.6 ± 3.8	45.6 ± 2.7	46.1 ± 3.1
129P3/J (R)	38.8 ± 4.5	47.7 ± 2.6	46.7 ± 3.9
Total (µg)			
A/J (S)	42.6 ± 3.8	1234.6 ± 115.0	905.0 ± 73.1
129P3/J (R)	38.8 ± 4.5	1198.1 ± 140.7	1233.7 ± 168.0

* Data obtained from eight collections per group of four animals, which were kept in pairs in metabolic cages. The means and standard deviations were obtained considering the values obtained for each of the eight weeks of treatment, for each experimental group.

**Table 2:** Mean (SD) maximum running speed (m/min) at baseline and at the end of the experiment and respective change in running speed.

Strain	Group	*n*	Mean (SD) Maximum Running Speed (m/min)		Change *
			Baseline	End of Experiment	Mean (SEM)
	SI (Control)	15	19.8 (6.0)	18.0 (5.4)	−1.8 (3.0) ^a^
A/J ^A^	SII (50 ppm F, no-exercise)	16	19.3 (6.6)	17.2 (5.7)	−2.0 (2.1) ^a^
	SIII (50 ppm F, exercise)	14	20.8 (5.5)	22.5 (4.5)	+1.7 (5.4) ^b^
	RI (Control)	15	24.8 (4.0)	21.8 (4.6)	−3.0 (3.2) ^a^
129P3/J ^A^	RII (50 ppm F, no-exercise)	15	24.2 (3.7)	22.0 (3.9)	−2.2 (3.3) ^a^
	RIII (50 ppm F, exercise)	15	25.0 (4.0)	26.6 (4.8)	+1.6 (4.4) ^b^

* “Change = speed at the end of the experiment − speed at baseline”. Similar uppercase superscripts indicate lack of significant difference between the strains for the change in running speed. Distinct lowercase superscripts indicate significant differences among the treatments for each strain. Two-way ANOVA followed by Bonferroni’s test (*p* < 0.05). *n* = 15.

**Table 3:** Mean (±SD) of plasma, bone ash (femur) and liver fluoride (F), glucose and insulin levels as well as HOMA2-IR (homeostasis model assessment 2 of insulin resistance) index, β-cell function (% B) and insulin sensitivity (% S) of A/J (susceptible to fluorosis; S) and 129P3/J (resistant to fluorosis; R) mice treated with water containing 50 ppm F or not (control; group I) and submitted to physical exercise (trained; group III) or not (sedentary; group II).

	Treatments		
Analysis	Control (I)	Sedentary + F (II)	Trained + F (III)
[F] plasma (µg/mL)			
A/J (S)	0.014 ± 0.004Aa	0.053 ± 0.030Bb	0.044 ± 0.015Bb
129P3/J (R)	0.017 ± 0.004Aa	0.086 ± 0.037Ab	0.089 ± 0.062Ab
[F] Femur (µg/Kg)			
A/J (S)	216.7 ± 60.1Aa	2024.0 ± 735.7Bb	2559.5 ± 1226.6Bb
129P3/J (R)	273.9 ± 172.9Aa	2914.7 ± 697.1Ab	3708.6 ± 1371.3Ac
[F] Liver (µg/g)			
A/J (S)	0.009 ± 0.001Aa	0.025 ± 0.004Ab	0.045 ± 0.008Bc
129P3/J (R)	0.009 ± 0.002Aa	0.023 ± 0.006Ab	0.103 ± 0.020Ac
Glucose (mg/dL)			
A/J (S)	176.7 ± 28.6A	174.2 ± 20.3A	168.1 ± 32.3A
129P3/J (R)	148.9 ± 28.3A	135.3 ± 32.2B *	145.5 ± 24.2A
Insulin (pmol/L)			
A/J (S)	65.8 ± 9.5	59.7 ± 6.7	65.8 ± 9.8
129P3/J (R)	58.7 ± 7.9	64.4 ± 12.5	60.9 ± 8.0
HOMA2-IR Index			
A/J (S)	1.41 ± 0.22	1.28 ± 0.16	1.30 ± 0.25
129P3/J (R)	1.22 ± 0.21	1.30 ± 0.27	1.25 ± 0.14
%B			
A/J (S)	32.7 ± 15.0A	29.4 ± 6.8A	33.2 ± 10.3A
129P3/J (R)	41.3 ± 15.3A	55.5 ± 25.9B **	48.2 ± 28.5A
%S			
A/J (S)	72.3 ± 9.9	79.3 ± 9.3	79.4 ± 14.1
129P3/J (R)	84.2 ± 14.1	79.8 ± 16.2	80.7 ± 9.7

For each variable, distinct uppercase letters in the same columns indicate significant differences between the strains and different lowercase letters in the same lines indicate significant differences among the treatments (2-way ANOVA and Bonferroni test for plasma). * and ** indicate Sidak and Tukey post-hoc tests, respectively. *p* < 0.05. *n* = 15. The physical exercise comprised a high-intensity interval training (HIIT) protocol on a treadmill, five days per week for eight weeks.

**Table 4:** Summary of the differences in expression and unique proteins found in the liver of A/J and 129P3/J mice, for each comparison.

Comparison	Total Number of Proteins Up or Down-Regulated *	Total Number of Unique Proteins in Each Group
SI vs. RI	83 up, 7 down	99 SI, 7 RI
SII vs. RII	52 up, 1 down	32 SII, 8 RII
SIII vs. RIII	26 up, 96 down	1 SIII, 90 RIII

* Up or down-regulation refers to the first term of each comparison. SI—A/J mice, deionized water, no-exercise; SII—A/J mice, water containing 50 ppm F, no-exercise; SIII—A/J mice, water containing 50 ppm F, exercise; RI—129P3/J mice, deionized water, no-exercise; RII—129P3/J mice, water containing 50 ppm F, no-exercise; RIII—129P3/J mice, water containing 50 ppm F, exercise.

**Table 5:** Summary of the main functions of proteins found in the liver in each comparison.

SI vs. RI	SII vs. RII	SIII vs. RIII
SI—↑ protein synthesis, energy flux and antioxidant enzymes.	SII—↑ protein synthesis, energy flux and antioxidant enzymes.	SIII—↓ protein synthesis, energy metabolism and detoxification; ↑ antioxidant enzymes.

Note: ↑ Increase and ↓ decrease.

**Table 6:** Summary of the differences in expression and unique proteins found in gastrocnemius of A/J and 129P3/J mice, for each comparison.

Comparison	Total Number of Proteins up or Down-Regulated *	Total Number of Unique Proteins in Each Group
SI vs. RI	5 up, 111 down	135 SI, 180 RI
SII vs. RII	99 up, 8 down	187 SII, 138 RII
SIII vs. RIII	85 up, 6 down	131 SIII, 99 RIII

* Up or down-regulation refers to the first term of each comparison. SI—A/J mice, deionized water, no-exercise; SII—A/J mice, water containing 50 ppm F, no-exercise; SIII—A/J mice, water containing 50 ppm F, exercise; RI—129P3/J mice, deionized water, no -exercise; RII—129P3/J mice, water containing 50 ppm F, no-exercise; RIII—129P3/J mice, water containing 50 ppm F, exercise.

**Table 7:** Summary of the main functions of proteins found in the gastrocnemius muscle in each comparison.

SI vs. RI	SII vs. RII	SIII vs. RIII
SI—↓ or absence proteins involved in muscle contraction and proteins related to the protein synthesis.	SII—↑ proteins related to muscle contraction/relaxation and proteins related to energy flux.	SIII—↑ or exclusivity proteins related to energy flux; ↑ proteins related to muscle contraction/relaxation.

Note: ↑ Increase and ↓ decrease.

## Data Availability

The data sets presented in this study can be found in online repositories. The names of the repository/repositories and accession number(s) can be found below: Peptide Atlas, accession PASS01723.

## Supplementary Materials

The following supporting information can be downloaded at: https://www.mdpi.com/article/10.3390/metabo12020117/s1, Table S1: Proteins with expression significantly altered in the liver of SI (A/J, deionized water, no-exercise) and RI (129P3/J, deionized water, no-exercise) mice, Table S2: Proteins with expression significantly altered in the liver of SII (A/J, water containing 50 ppm F, no-exercise) and RII (129P3/J, water containing 50 ppm F, no-exercise) mice, Table S3: Proteins with expression significantly altered in the liver of SIII (A/J, water containing 50 ppm F, exercise) and RIII (129P3/J, water containing 50 ppm F, exercise) mice, Table S4: Proteins with expression significantly altered in the gastrocnemius of SI (A/J, deionized water, no-exercise) and RI (129P3/J, deionized water, no-exercise) mice, Table S5: Proteins with expression significantly altered in the gastrocnemius of SII (A/J, water containing 50 ppm F, no-exercise) and RII (129P3/J, water containing 50 ppm F, no-exercise) mice, Table S6: Proteins with expression significantly altered in the gastrocnemius of SIII (A/J, water containing 50 ppm F, exercise) and RIII (129P3/J, water containing 50 ppm F, exercise) mice.
