# Supplementary material for: Effect of Physical Exercise and Genetic Background on Glucose Homeostasis and Liver/Muscle Proteomes in Mice
Source: Metabolites. 2022 Jan 25;12(2):117. doi: 10.3390/metabo12020117 (PMC8878675; doi:10.3390/metabo12020117)
Supplement: Supplementary file 1 [file metabolites-12-00117-s001.zip › metabolites-1575892-supplementary.pdf]

Table S1. Proteins with expression significantly altered in the liver of SI (A/J, deionized water, no-exercise) and RI (129P3/J, deionized water, no-exercise) mice

[a]Acession number	Protein name	PLGS Score	[b]*Ratio* SI:RI
Q8VCH0	3-ketoacyl-CoA thiolase B_ peroxisomal	68	4.31
P10648	Glutathione S-transferase A2	527	2.92
O08807	Peroxiredoxin-4	70	2.86
Q921H8	3-ketoacyl-CoA thiolase A_ peroxisomal	111	2.77
P30115	Glutathione S-transferase A3	527	2.72
P13745	Glutathione S-transferase A1	527	2.69
Q6P8Q0	Glutathione S-transferase	527	2.69
P62631	Elongation factor 1-alpha 2	56	2.64
P49429	4-hydroxyphenylpyruvate dioxygenase	205	2.59
P16015	Carbonic anhydrase 3	558	2.32
P24472	Glutathione S-transferase A4	30	2.16
P0C0S6	Histone H2A.Z	246	2.12
C0HKE2	Histone H2A type 1-C	246	2.10
C0HKE9	Histone H2A type 1-P	246	2.10
C0HKE3	Histone H2A type 1-D	246	2.08
C0HKE5	Histone H2A type 1-G	246	2.08
Q64523	Histone H2A type 2-C	246	2.08
Q8BFU2	Histone H2A type 3	246	2.08
Q8R1M2	Histone H2A.J	246	2.08
Q3THW5	Histone H2A.V	246	2.08
C0HKE1	Histone H2A type 1-B	246	2.05
C0HKE4	Histone H2A type 1-E	246	2.05
C0HKE6	Histone H2A type 1-I	246	2.05
C0HKE8	Histone H2A type 1-O	246	2.05
Q6GSS7	Histone H2A type 2-A	246	2.05
Q64522	Histone H2A type 2-B	246	2.05
P62806	Histone H4	265	2.05
Q8CGP5	Histone H2A type 1-F	246	2.03
Q8CGP7	Histone H2A type 1-K	246	2.03
C0HKE7	Histone H2A type 1-N	246	2.03
P27661	Histone H2AX	246	2.03
Q8CGP6	Histone H2A type 1-H	246	2.01
P11352	Glutathione peroxidase 1	441	1.90
Q9JHW9	Aldehyde dehydrogenase family 1 member A3	46	1.70
P50247	Adenosylhomocysteinase	327	1.68
Q9CZS1	Aldehyde dehydrogenase X_ mitochondrial	46	1.68
Q9QXD6	Fructose-1_6-bisphosphatase 1	261	1.65
Q62148	Retinal dehydrogenase 2	46	1.65
O35945	Aldehyde dehydrogenase_ cytosolic 1	241	1.63
P24549	Retinal dehydrogenase 1	317	1.63
P47738	Aldehyde dehydrogenase_ mitochondrial	237	1.62
Q99LC5	Electron transfer flavoprotein subunit alpha_ mitochondrial	114	1.62
P24270	Catalase	342	1.58
P35700	Peroxiredoxin-1	284	1.58
Q9DCW4	Electron transfer flavoprotein subunit beta	236	1.55

P14152	Malate dehydrogenase_ cytoplasmic	230	1.54
P08228	Superoxide dismutase [Cu-Zn]	123	1.52
P54869	Hydroxymethylglutaryl-CoA synthase_ mitochondrial	185	1.49
Q64433	10 kDa heat shock protein_ mitochondrial	333	1.43
P05202	Aspartate aminotransferase_ mitochondrial	179	1.42
Q64374	Regucalcin	353	1.42
P26443	Glutamate dehydrogenase 1_ mitochondrial	154	1.36
P11725	Ornithine carbamoyltransferase_ mitochondrial	183	1.36
P52760	2-iminobutanoate/2-iminopropanoate deaminase	221	1.35
P12710	Fatty acid-binding protein_ liver	2940	1.35
P15626	Glutathione S-transferase Mu 2	582	1.35
Q61176	Arginase-1	956	1.34
Q8R5I6	Glutathione S-transferase mu 4	582	1.34
Q80W21	Glutathione S-transferase Mu 7	582	1.34
Q03265	ATP synthase subunit alpha_ mitochondrial	293	1.32
F6Y363	Uncharacterized protein	582	1.32
P16460	Argininosuccinate synthase	570	1.31
Q8BFZ3	Beta-actin-like protein 2	386	1.31
P08249	Malate dehydrogenase_ mitochondrial	286	1.31
P56480	ATP synthase subunit beta_ mitochondrial	352	1.30
Q8BWT1	3-ketoacyl-CoA thiolase_ mitochondrial	301	1.28
P10126	Elongation factor 1-alpha 1	238	1.28
P70694	Estradiol 17 beta-dehydrogenase 5	411	1.28
P48774	Glutathione S-transferase Mu 5	87	1.28
Q9QXF8	Glycine N-methyltransferase	274	1.28
P63260	Actin_ cytoplasmic 2	1328	1.26
Q91Y97	Fructose-bisphosphate aldolase B	301	1.23
P68033	Actin_ alpha cardiac muscle 1	1147	1.22
P68134	Actin_ alpha skeletal muscle	1147	1.22
P62737	Actin_ aortic smooth muscle	1147	1.22
P60710	Actin_ cytoplasmic 1	1328	1.22
P63268	Actin_ gamma-enteric smooth muscle	1147	1.22
P10649	Glutathione S-transferase Mu 1	368	1.19
P16858	Glyceraldehyde-3-phosphate dehydrogenase	493	1.17
O35490	Betaine--homocysteine S-methyltransferase 1	1350	1.15
P25688	Uricase	589	1.13
Q8C196	Carbamoyl-phosphate synthase [ammonia]_ mitochondrial	1600	1.12
P02089	Hemoglobin subunit beta-2	3090	1.06
P02088	Hemoglobin subunit beta-1	4320	0.90
P19157	Glutathione S-transferase P 1	396	0.89
Q91VB8	Alpha globin 1	2856	0.89
P01942	Hemoglobin subunit alpha	2604	0.89
P02104	Hemoglobin subunit epsilon-Y2	2045	0.89
P46425	Glutathione S-transferase P 2	101	0.79
V9GXQ2	Uncharacterized protein	183	0.66
Q9CQ62	2_4-dienoyl-CoA reductase_ mitochondrial	214	SI*
Q78JT3	3-hydroxyanthranilate 3_4-dioxygenase	651	SI
P47955	60S acidic ribosomal protein P1	824	SI
Q99KI0	Aconitate hydratase_ mitochondrial	83	SI

P31786	Acyl-CoA-binding protein	2938	SI
Q91VA0	Acyl-coenzyme A synthetase ACSM1_ mitochondrial	232	SI
Q64437	Alcohol dehydrogenase class 4 mu/sigma chain	197	SI
P28474	Alcohol dehydrogenase class-3	1575	SI
Q8BH00	Aldehyde dehydrogenase family 8 member A1	166	SI
Q9DBF1	Alpha-aminoadipic semialdehyde dehydrogenase	271	SI
P00687	Alpha-amylase 1	25	SI
P17182	Alpha-enolase	954	SI
P05201	Aspartate aminotransferase_ cytoplasmic	461	SI
P21550	Beta-enolase	219	SI
P34914	Bifunctional epoxide hydrolase 2	835	SI
Q8VCT4	Carboxylesterase 1D	216	SI
Q91WU0	Carboxylesterase 1F	188	SI
Q63880	Carboxylesterase 3A	235	SI
Q99KF0	Caspase recruitment domain-containing protein 14	28	SI
P56395	Cytochrome b5	772	SI
Q9CZ13	Cytochrome b-c1 complex subunit 1_ mitochondrial	214	SI
P19783	Cytochrome c oxidase subunit 4 isoform 1_ mitochondrial	518	SI
P12787	Cytochrome c oxidase subunit 5A_ mitochondrial	561	SI
Q64458	Cytochrome P450 2C29	222	SI
P28271	Cytoplasmic aconitate hydratase	84	SI
O35215	D-dopachrome decarboxylase	3765	SI
Q8CHT0	Delta-1-pyrroline-5-carboxylate dehydrogenase_ mitochondrial	463	SI
Q3U319	E3 ubiquitin-protein ligase BRE1B	26	SI
Q8BL66	Early endosome antigen 1	17	SI
P42125	Enoyl-CoA delta isomerase 1_ mitochondrial	283	SI
P19096	Fatty acid synthase	36	SI
Q91XD4	Formimidoyltransferase-cyclodeaminase	247	SI
P35505	Fumarylacetoacetase	980	SI
Q64467	Glyceraldehyde-3-phosphate dehydrogenase_ testis-specific	109	SI
Q8CBB6	Histone H2B	3067	SI
P70696	Histone H2B type 1-A	1718	SI
Q64475	Histone H2B type 1-B	3067	SI
Q6ZWY9	Histone H2B type 1-C/E/G	3067	SI
P10853	Histone H2B type 1-F/J/L	3067	SI
Q64478	Histone H2B type 1-H	3067	SI
Q8CGP1	Histone H2B type 1-K	3067	SI
P10854	Histone H2B type 1-M	3067	SI
Q8CGP2	Histone H2B type 1-P	3067	SI
Q64525	Histone H2B type 2-B	3067	SI
Q64524	Histone H2B type 2-E	2684	SI
Q9D2U9	Histone H2B type 3-A	2684	SI
Q8CGP0	Histone H2B type 3-B	2684	SI
O88844	Isocitrate dehydrogenase [NADP] cytoplasmic	250	SI
P54071	Isocitrate dehydrogenase [NADP]_ mitochondrial	174	SI
Q9CPU0	Lactoylglutathione lyase	944	SI
P41216	Long-chain-fatty-acid--CoA ligase 1	572	SI
P11588	Major urinary protein 1	389	SI
P04938	Major urinary protein 11	389	SI

P11589	Major urinary protein 2	389	SI
P11591	Major urinary protein 5	98	SI
P02762	Major urinary protein 6	389	SI
Q9JM52	Misshapen-like kinase 1	86	SI
Q8K009	Mitochondrial 10-formyltetrahydrofolate dehydrogenase	199	SI
P97820	Mitogen-activated protein kinase kinase kinase kinase 4	96	SI
Q8BZW8	NHL repeat-containing protein 2	37	SI
P32020	Non-specific lipid-transfer protein	253	SI
F6ZDS4	Nucleoprotein TPR	23	SI
P29758	Ornithine aminotransferase_ mitochondrial	318	SI
P17742	Peptidyl-prolyl cis-trans isomerase A	420	SI
O08709	Peroxiredoxin-6	457	SI
Q9DBM2	Peroxisomal bifunctional enzyme	101	SI
P09411	Phosphoglycerate kinase 1	136	SI
P09041	Phosphoglycerate kinase 2	78	SI
Q8VCR7	Protein ABHD14B	588	SI
P27773	Protein disulfide-isomerase A3	209	SI
P09103	Protein disulfide-isomerase	222	SI
O88451	Retinol dehydrogenase 7	110	SI
P17563	Selenium-binding protein 1	946	SI
Q63836	Selenium-binding protein 2	940	SI
Q07417	Short-chain specific acyl-CoA dehydrogenase_ mitochondrial	246	SI
Q64442	Sorbitol dehydrogenase	347	SI
P38647	Stress-70 protein_ mitochondrial	80	SI
P52196	Thiosulfate sulfurtransferase	231	SI
B9EKN8	TRAF2 and NCK interacting kinase	86	SI
P83510	Traf2 and NCK-interacting protein kinase	86	SI
Q01853	Transitional endoplasmic reticulum ATPase	45	SI
P40142	Transketolase	76	SI
Q8BMS1	Trifunctional enzyme subunit alpha_ mitochondrial	107	SI
Q8VC30	Triokinase/FMN cyclase	209	SI
P17751	Triosephosphate isomerase	1172	SI
P68369	Tubulin alpha-1A chain	299	SI
P05213	Tubulin alpha-1B chain	299	SI
P68373	Tubulin alpha-1C chain	299	SI
P05214	Tubulin alpha-3 chain	247	SI
P68368	Tubulin alpha-4A chain	247	SI
Q9JJZ2	Tubulin alpha-8 chain	247	SI
Q9ERD7	Tubulin beta-3 chain	47	SI
Q9D6F9	Tubulin beta-4A chain	152	SI
P68372	Tubulin beta-4B chain	144	SI
P99024	Tubulin beta-5 chain	47	SI
E9Q3T0	Uncharacterized protein	685	SI
Q8VC12	Urocanate hydratase	38	SI
Q8R164	Valacyclovir hydrolase	179	SI
Q80VW5	Whirlin	22	SI
P05977	Myosin light chain 1/3_ skeletal muscle isoform	452	RI
Q5SX40	Myosin-1	96	RI
Q5SX39	Myosin-4	105	RI

Q02566	Myosin-6	63	RI
Q91Z83	Myosin-7	63	RI
P13542	Myosin-8	96	RI
Q99LX0	Protein DJ-1	172	RI

[a]Identification is based on proteins ID from UniProt protein database, reviewed only (http://www.uniprot.org/).
[b]Proteins with expression significantly altered are organized according to the ratio.
*Indicates unique proteins in alphabetical order.

Table S2. Proteins with expression significantly altered in the liver of SII (A/J, water containing 50 ppm F, no-exercise) and RII (129P3/J, water containing 50 ppm F, no-exercise) mice

[a]Acession number	Protein name	PLGS Score	[b]*Ratio* SII:RII
V9GXA7	Uncharacterized protein	74	3.35
V9GXQ2	Uncharacterized protein	35	2.89
P16015	Carbonic anhydrase 3	402	2.64
Q9QXD6	Fructose-1_6-bisphosphatase 1	236	2.03
P16460	Argininosuccinate synthase	195	1.97
P02088	Hemoglobin subunit beta-1	3980	1.97
P02104	Hemoglobin subunit epsilon-Y2	1376	1.95
Q91VB8	Alpha globin 1	4327	1.84
P16858	Glyceraldehyde-3-phosphate dehydrogenase	641	1.79
P02089	Hemoglobin subunit beta-2	2526	1.77
B2RQC6	CAD protein	8	1.68
P50247	Adenosylhomocysteinase	270	1.60
P24549	Retinal dehydrogenase 1	175	1.58
P11352	Glutathione peroxidase 1	626	1.57
P07724	Serum albumin	245	1.55
O35945	Aldehyde dehydrogenase_ cytosolic 1	143	1.52
P00329	Alcohol dehydrogenase 1	180	1.51
P08249	Malate dehydrogenase_ mitochondrial	140	1.48
P24270	Catalase	351	1.46
Q99LC5	Electron transfer flavoprotein subunit alpha_ mitochondrial	111	1.46
P01942	Hemoglobin subunit alpha	4327	1.46
P10126	Elongation factor 1-alpha 1	475	1.40
Q9JHW9	Aldehyde dehydrogenase family 1 member A3	37	1.36
P35700	Peroxiredoxin-1	153	1.35
Q62148	Retinal dehydrogenase 2	37	1.35
P70694	Estradiol 17 beta-dehydrogenase 5	137	1.34
P12710	Fatty acid-binding protein_ liver	3193	1.34
P54869	Hydroxymethylglutaryl-CoA synthase_ mitochondrial	165	1.32
P05202	Aspartate aminotransferase_ mitochondrial	237	1.30
Q8BWT1	3-ketoacyl-CoA thiolase_ mitochondrial	319	1.28
P63268	Actin_ gamma-enteric smooth muscle	1730	1.26
P47738	Aldehyde dehydrogenase_ mitochondrial	226	1.26
Q9DCW4	Electron transfer flavoprotein subunit beta	349	1.26
P68033	Actin_ alpha cardiac muscle 1	1730	1.25
P62737	Actin_ aortic smooth muscle	1730	1.25
Q8C196	Carbamoyl-phosphate synthase [ammonia]_ mitochondrial	1655	1.25
P68134	Actin_ alpha skeletal muscle	1730	1.23

P56480	ATP synthase subunit beta_ mitochondrial	390	1.23
P11725	Ornithine carbamoyltransferase_ mitochondrial	206	1.23
Q64374	Regucalcin	361	1.23
Q03265	ATP synthase subunit alpha_ mitochondrial	299	1.21
P25688	Uricase	526	1.21
P60710	Actin_ cytoplasmic 1	2007	1.19
O35490	Betaine--homocysteine S-methyltransferase 1	876	1.19
P26443	Glutamate dehydrogenase 1_ mitochondrial	330	1.19
P10649	Glutathione S-transferase Mu 1	790	1.19
P63260	Actin_ cytoplasmic 2	2007	1.16
Q91Y97	Fructose-bisphosphate aldolase B	277	1.16
Q8BFZ3	Beta-actin-like protein 2	428	1.16
Q91WS4	S-methylmethionine--homocysteine S- methyltransferase BHMT2	485	1.14
P19157	Glutathione S-transferase P 1	1249	1.13
Q9QXF8	Glycine N-methyltransferase	467	1.12
P46425	Glutathione S-transferase P 2	491	0.61
P49429	4-hydroxyphenylpyruvate dioxygenase	220	SII*
P31786	Acyl-CoA-binding protein	592	SII
Q8VCT4	Carboxylesterase 1D	84	SII
O35215	D-dopachrome decarboxylase	206	SII
P62631	Elongation factor 1-alpha 2	34	SII
P13745	Glutathione S-transferase A1	355	SII
P10648	Glutathione S-transferase A2	331	SII
P30115	Glutathione S-transferase A3	355	SII
P24472	Glutathione S-transferase A4	25	SII
P15626	Glutathione S-transferase Mu 2	408	SII
P48774	Glutathione S-transferase Mu 5	212	SII
Q80W21	Glutathione S-transferase Mu 7	416	SII
Q64467	Glyceraldehyde-3-phosphate dehydrogenase_ testis-specific		SII
Q8CBB6	Histone H2B	590	SII
P70696	Histone H2B type 1-A	391	SII
Q64475	Histone H2B type 1-B	590	SII
Q6ZWY9	Histone H2B type 1-C/E/G	590	SII
P10853	Histone H2B type 1-F/J/L	590	SII
Q64478	Histone H2B type 1-H	590	SII
Q8CGP1	Histone H2B type 1-K	590	SII
P10854	Histone H2B type 1-M	590	SII
Q8CGP2	Histone H2B type 1-P	590	SII
Q64525	Histone H2B type 2-B	590	SII
Q64524	Histone H2B type 2-E	590	SII
Q9D2U9	Histone H2B type 3-A	590	SII
Q8CGP0	Histone H2B type 3-B	590	SII
P62806	Histone H4	161	SII
P06151	L-lactate dehydrogenase A chain	116	SII
P14152	Malate dehydrogenase_ cytoplasmic	376	SII
O08807	Peroxiredoxin-4	119	SII
O08709	Peroxiredoxin-6	36	SII
F6Y363	Uncharacterized protein	408	SII
P11588	Major urinary protein 1	849	RII

P04938	Major urinary protein 11	849	RII
B5X0G2	Major urinary protein 17	849	RII
A2BIM8	Major urinary protein 18	849	RII
P11589	Major urinary protein 2	849	RII
P11591	Major urinary protein 5	811	RII
P02762	Major urinary protein 6	849	RII
P32020	Non-specific lipid-transfer protein	82	RII

[a]Identification is based on proteins ID from UniProt protein database, reviewed only (http://www.uniprot.org/).
[b]Proteins with expression significantly altered are organized according to the ratio.
*Indicates unique proteins in alphabetical order.

Table S3. Proteins with expression significantly altered in the liver of SIII (A/J, water containing 50 ppm F, exercise) and RIII (129P3/J, water containing 50 ppm F, exercise) mice

[a]**Acession number**	**Protein name**	**PLGS Score**	[b]***Ratio* SIII:RIII**
P16015	Carbonic anhydrase 3	3069	2.94
P12710	Fatty acid-binding protein_ liver	21785	2.05
Q61176	Arginase-1	4188	1.82
O35945	Aldehyde dehydrogenase_ cytosolic 1	1018	1.79
Q8C196	Carbamoyl-phosphate synthase [ammonia]_ mitochondrial	8837	1.77
P02088	Hemoglobin subunit beta-1	27458	1.67
P54869	Hydroxymethylglutaryl-CoA synthase_ mitochondrial	1254	1.67
O35490	Betaine--homocysteine S-methyltransferase 1	5948	1.63
P24549	Retinal dehydrogenase 1	1374	1.63
P16858	Glyceraldehyde-3-phosphate dehydrogenase	2946	1.62
Q9CZS1	Aldehyde dehydrogenase X_ mitochondrial	89	1.58
P02104	Hemoglobin subunit epsilon-Y2	1854	1.57
Q9JHW9	Aldehyde dehydrogenase family 1 member A3	89	1.55
Q62148	Retinal dehydrogenase 2	89	1.49
Q91Y97	Fructose-bisphosphate aldolase B	3833	1.38
O35215	D-dopachrome decarboxylase	1061	1.32
P10649	Glutathione S-transferase Mu 1	12177	1.28
P11725	Ornithine carbamoyltransferase_ mitochondrial	1289	1.28
P08228	Superoxide dismutase [Cu-Zn]	1918	1.28
P26443	Glutamate dehydrogenase 1_ mitochondrial	1955	1.27
P02089	Hemoglobin subunit beta-2	9125	1.21
Q91WS4	S-methylmethionine--homocysteine S- methyltransferase BHMT2	1884	1.13
P68134	Actin_ alpha skeletal muscle	8945	1.12
P68033	Actin_ alpha cardiac muscle 1	8949	1.09
P62737	Actin_ aortic smooth muscle	8945	1.09
P63268	Actin_ gamma-enteric smooth muscle	8945	1.09
P01942	Hemoglobin subunit alpha	11151	0.95
P60710	Actin_ cytoplasmic 1	10876	0.94
Q8BFZ3	Beta-actin-like protein 2	2497	0.80
P52760	2-iminobutanoate/2-iminopropanoate deaminase	799	0.79
Q05920	Pyruvate carboxylase_ mitochondrial	128	0.77
P05202	Aspartate aminotransferase_ mitochondrial	1309	0.76
Q91XD4	Formimidoyltransferase-cyclodeaminase	494	0.75
P63038	60 kDa heat shock protein_ mitochondrial	306	0.73

Q99LB7	Sarcosine dehydrogenase_ mitochondrial	285	0.71
P99029	Peroxiredoxin-5_ mitochondrial	548	0.70
Q8VCN5	Cystathionine gamma-lyase	507	0.69
Q99LC5	Electron transfer flavoprotein subunit alpha_ mitochondrial	466	0.68
Q9DCW4	Electron transfer flavoprotein subunit beta	3282	0.68
P25688	Uricase	3095	0.68
P08249	Malate dehydrogenase_ mitochondrial	1105	0.67
Q8VCT4	Carboxylesterase 1D	240	0.66
P07724	Serum albumin	1088	0.65
Q8CGP5	Histone H2A type 1-F	921	0.64
Q64433	10 kDa heat shock protein_ mitochondrial	1588	0.64
P12787	Cytochrome c oxidase subunit 5A_ mitochondrial	648	0.64
Q9CQ62	2_4-dienoyl-CoA reductase_ mitochondrial	68	0.63
A0A0N4SVE	Uncharacterized protein	2588	0.63
P11679	Keratin_ type II cytoskeletal 8	207	0.63
Q64522	Histone H2A type 2-B	921	0.62
Q03265	ATP synthase subunit alpha_ mitochondrial	1776	0.62
B2RQC6	CAD protein	58	0.62
Q8R1M2	Histone H2A.J	921	0.61
P14152	Malate dehydrogenase_ cytoplasmic	1349	0.61
P31786	Acyl-CoA-binding protein	1514	0.61
P00329	Alcohol dehydrogenase 1	1008	0.61
Q64523	Histone H2A type 2-C	921	0.61
P0C0S6	Histone H2A.Z	921	0.61
P27661	Histone H2AX	921	0.61
O09173	Homogentisate 1_2-dioxygenase	232	0.61
C0HKE3	Histone H2A type 1-D	921	0.60
C0HKE6	Histone H2A type 1-I	921	0.60
Q6GSS7	Histone H2A type 2-A	921	0.60
Q3THW5	Histone H2A.V	921	0.60
Q8K009	Mitochondrial 10-formyltetrahydrofolate dehydrogenase	133	0.60
C0HKE8	Histone H2A type 1-O	921	0.59
P20029	78 kDa glucose-regulated protein	633	0.59
P56480	ATP synthase subunit beta_ mitochondrial	2012	0.59
Q9QXD6	Fructose-1_6-bisphosphatase 1	1052	0.59
C0HKE1	Histone H2A type 1-B	921	0.59
C0HKE2	Histone H2A type 1-C	921	0.59
C0HKE5	Histone H2A type 1-G	921	0.59
Q8CGP6	Histone H2A type 1-H	921	0.59
Q8CGP7	Histone H2A type 1-K	921	0.59
C0HKE9	Histone H2A type 1-P	921	0.59
P62806	Histone H4	801	0.59
V9GXA7	Uncharacterized protein	771	0.59
Q78JT3	3-hydroxyanthranilate 3_4-dioxygenase	493	0.59
P47738	Aldehyde dehydrogenase_ mitochondrial	1659	0.59
Q8BFU2	Histone H2A type 3	921	0.58
C0HKE4	Histone H2A type 1-E	921	0.58
C0HKE7	Histone H2A type 1-N	921	0.58

Q8BWT1	3-ketoacyl-CoA thiolase_ mitochondrial	1849	0.58
Q9EQ20	Methylmalonate-semialdehyde dehydrogenase[acylating]_ mitochondrial	271	0.57
P05201	Aspartate aminotransferase_ cytoplasmic	382	0.56
Q8R0Y6	Cytosolic 10-formyltetrahydrofolate dehydrogenase	3301	0.56
Q64475	Histone H2B type 1-B	1475	0.56
P10853	Histone H2B type 1-F/J/L	1475	0.56
Q64478	Histone H2B type 1-H	1475	0.56
Q8CGP1	Histone H2B type 1-K	1475	0.56
Q8CGP2	Histone H2B type 1-P	1475	0.56
Q64525	Histone H2B type 2-B	1475	0.56
Q9D2U9	Histone H2B type 3-A	1155	0.56
P24270	Catalase	3316	0.55
P11352	Glutathione peroxidase 1	2205	0.55
Q6ZWY9	Histone H2B type 1-C/E/G	1475	0.55
P10854	Histone H2B type 1-M	1475	0.55
Q64524	Histone H2B type 2-E	1155	0.55
Q8CGP0	Histone H2B type 3-B	1155	0.55
P49429	4-hydroxyphenylpyruvate dioxygenase	1665	0.55
P35700	Peroxiredoxin-1	2693	0.55
P70696	Histone H2B type 1-A	587	0.54
Q01768	Nucleoside diphosphate kinase B	584	0.53
Q91X83	S-adenosylmethionine synthase isoform type-1	1571	0.53
P70694	Estradiol 17 beta-dehydrogenase 5	2293	0.52
P19157	Glutathione S-transferase P 1	5065	0.52
P56395	Cytochrome b5	998	0.51
P32020	Non-specific lipid-transfer protein	522	0.47
P35505	Fumarylacetoacetase	812	0.46
P50247	Adenosylhomocysteinase	5869	0.46
Q8QZT1	Acetyl-CoA acetyltransferase_ mitochondrial	959	0.45
P16460	Argininosuccinate synthase	2388	0.44
P62631	Elongation factor 1-alpha 2	427	0.43
O08807	Peroxiredoxin-4	518	0.42
F6Y363	Uncharacterized protein	3705	0.41
P15626	Glutathione S-transferase Mu 2	3705	0.41
Q80W21	Glutathione S-transferase Mu 7	3705	0.41
Q9QXF8	Glycine N-methyltransferase	3693	0.41
P48774	Glutathione S-transferase Mu 5	163	0.40
P13745	Glutathione S-transferase A1	2794	0.37
P10648	Glutathione S-transferase A2	2747	0.37
P30115	Glutathione S-transferase A3	3206	0.37
Q9CPU0	Lactoylglutathione lyase	407	SIII*
Q921H8	3-ketoacyl-CoA thiolase A_ peroxisomal	613	RIII
Q8VCH0	3-ketoacyl-CoA thiolase B_ peroxisomal	613	RIII
P14206	40S ribosomal protein SA	192	RIII
P47955	60S acidic ribosomal protein P1	1191	RIII
Q91WG0	Acylcarnitine hydrolase	17	RIII
Q91VA0	Acyl-coenzyme A synthetase ACSM1_ mitochondrial	64	RIII
Q8QZR5	Alanine aminotransferase 1	102	RIII

P28474	Alcohol dehydrogenase class-3	1777	RIII
Q8BH00	Aldehyde dehydrogenase family 8 member A1	77	RIII
Q9DBF1	Alpha-aminoadipic semialdehyde dehydrogenase	423	RIII
P17182	Alpha-enolase	769	RIII
P21550	Beta-enolase	71	RIII
P34914	Bifunctional epoxide hydrolase 2	438	RIII
Q8VCU1	Carboxylesterase 3B	89	RIII
P19783	Cytochrome c oxidase subunit 4 isoform 1_ mitochondrial	245	RIII
Q8CHT0	Delta-1-pyrroline-5-carboxylate dehydrogenase_ mitochondrial	328	RIII
Q8BVI4	Dihydropteridine reductase	368	RIII
Q9DBT9	Dimethylglycine dehydrogenase_ mitochondrial	50	RIII
Q8BH95	Enoyl-CoA hydratase_ mitochondrial	105	RIII
P19096	Fatty acid synthase	25	RIII
P15105	Glutamine synthetase	391	RIII
P24472	Glutathione S-transferase A4	35	RIII
Q64467	Glyceraldehyde-3-phosphate dehydrogenase_ testis-specific	45	RIII
Q61696	Heat shock 70 kDa protein 1A	157	RIII
P17879	Heat shock 70 kDa protein 1B	157	RIII
P16627	Heat shock 70 kDa protein 1-like	157	RIII
P63017	Heat shock cognate 71 kDa protein	741	RIII
Q9CQN1	Heat shock protein 75 kDa_ mitochondrial	187	RIII
P07901	Heat shock protein HSP 90-alpha	218	RIII
P11499	Heat shock protein HSP 90-beta	510	RIII
P17156	Heat shock-related 70 kDa protein 2	250	RIII
P40936	Indolethylamine N-methyltransferase	267	RIII
Q9D819	Inorganic pyrophosphatase	254	RIII
O88844	Isocitrate dehydrogenase [NADP] cytoplasmic	99	RIII
P05784	Keratin_ type I cytoskeletal 18	115	RIII
Q99M73	Keratin_ type II cuticular Hb4	7	RIII
P06151	L-lactate dehydrogenase A chain	518	RIII
P16125	L-lactate dehydrogenase B chain	293	RIII
P00342	L-lactate dehydrogenase C chain	293	RIII
P41216	Long-chain-fatty-acid--CoA ligase 1	375	RIII
P11588	Major urinary protein 1	1114	RIII
P04938	Major urinary protein 11	1114	RIII
B5X0G2	Major urinary protein 17	1114	RIII
A2BIM8	Major urinary protein 18	1114	RIII
P11589	Major urinary protein 2	1114	RIII
P11591	Major urinary protein 5	1114	RIII
P02762	Major urinary protein 6	1114	RIII
Q8R4H7	N-acetylglutamate synthase_ mitochondrial	207	RIII
P17742	Peptidyl-prolyl cis-trans isomerase A	967	RIII
P24369	Peptidyl-prolyl cis-trans isomerase B	111	RIII
O08709	Peroxiredoxin-6	710	RIII
P51660	Peroxisomal multifunctional enzyme type 2	130	RIII
P70296	Phosphatidylethanolamine-binding protein 1	506	RIII
P09411	Phosphoglycerate kinase 1	124	RIII
P09041	Phosphoglycerate kinase 2	82	RIII
Q8VCR7	Protein ABHD14B	353	RIII

P09103	Protein disulfide-isomerase	144	RIII
P27773	Protein disulfide-isomerase A3	90	RIII
Q99LX0	Protein DJ-1	506	RIII
Q8C627	Protein FAM221B	103	RIII
O55125	Protein NipSnap homolog 1	560	RIII
Q2PZL6	Protocadherin Fat 4	15	RIII
Q8QZR3	Pyrethroid hydrolase Ces2a	104	RIII
Q3THS6	S-adenosylmethionine synthase isoform type-2	309	RIII
Q99J08	SEC14-like protein 2	260	RIII
P17563	Selenium-binding protein 1	442	RIII
Q63836	Selenium-binding protein 2	423	RIII
Q9R0P3	S-formylglutathione hydrolase	331	RIII
Q64442	Sorbitol dehydrogenase	646	RIII
P38647	Stress-70 protein_ mitochondrial	129	RIII
Q8R086	Sulfite oxidase_ mitochondrial	54	RIII
Q01853	Transitional endoplasmic reticulum ATPase	134	RIII
Q8BMS1	Trifunctional enzyme subunit alpha_ mitochondrial	290	RIII
Q99JY0	Trifunctional enzyme subunit beta_ mitochondrial	80	RIII
P17751	Triosephosphate isomerase	1762	RIII
Q9R1R2	Tripartite motif-containing protein 3	153	RIII
Q3UX10	Tubulin alpha chain-like 3	10	RIII
P68369	Tubulin alpha-1A chain	377	RIII
P05213	Tubulin alpha-1B chain	381	RIII
P68373	Tubulin alpha-1C chain	377	RIII
P05214	Tubulin alpha-3 chain	236	RIII
P68368	Tubulin alpha-4A chain	241	RIII
Q9JJZ2	Tubulin alpha-8 chain	236	RIII
Q7TMM9	Tubulin beta-2A chain	333	RIII
Q9CWF2	Tubulin beta-2B chain	314	RIII
Q9ERD7	Tubulin beta-3 chain	230	RIII
Q9D6F9	Tubulin beta-4A chain	447	RIII
P68372	Tubulin beta-4B chain	470	RIII
P99024	Tubulin beta-5 chain	470	RIII
E9Q3T0	Uncharacterized protein	560	RIII

[a]Identification is based on proteins ID from UniProt protein database, reviewed only (http://www.uniprot.org/).
[b]Proteins with expression significantly altered are organized according to the ratio.
*Indicates unique proteins in alphabetical order.

Table S4. Proteins with expression significantly altered in the gastrocnemius of SI (A/J, deionized water, no-exercise) and RI (129P3/J, deionized water, no-exercise) mice

[a]**Acession number**	**Protein name**	**PLGS Score**	[b]***Ratio*** **SI:RI**
Q62388	Serine-protein kinase ATM	92	2.18
Q7TMW6	Cytosolic Fe-S cluster assembly factor	97	2.10
Q60974	Nuclear receptor corepressor 1	110	1.58
Q08481	Platelet endothelial cell adhesion molecule	68	1.57
Q8R429	Sarcoplasmic/endoplasmic reticulum calcium ATPase 1	1310	1.07
Q9WUB3	Glycogen phosphorylase_ muscle form	4719	0.98
P58771	Tropomyosin alpha-1 chain	10907	0.90

P52480	Pyruvate kinase	9371	0.84
P21107	Tropomyosin alpha-3 chain	526	0.81
P07310	Creatine kinase M-type	20796	0.80
Q3TJD7	PDZ and LIM domain protein 7	1073	0.80
P13412	Troponin I_ fast skeletal muscle	1865	0.80
Q7TQ48	Sarcalumenin	57	0.79
P05202	Aspartate aminotransferase_ mitochondrial	238	0.79
P97457	Myosin regulatory light chain 2_ skeletal muscle isoform	7565	0.77
Q99KI0	Aconitate hydratase_ mitochondrial	170	0.76
P47857	ATP-dependent 6-phosphofructokinase_ muscle type	551	0.76
A2AQP0	Myosin-7B	1899	0.76
Q8VDD5	Myosin-9	314	0.76
Q9R0Y5	Adenylate kinase isoenzyme 1	6335	0.76
Q62234	Myomesin-1	185	0.76
Q6URW6	Myosin-14	309	0.76
Q9QZ47	Troponin T_ fast skeletal muscle	1883	0.76
O09165	Calsequestrin-1	1739	0.75
O08638	Myosin-11	319	0.75
P60710	Actin_ cytoplasmic 1	78650	0.74
P63260	Actin_ cytoplasmic 2	78650	0.74
Q61879	Myosin-10	309	0.74
Q5XKE0	Myosin-binding protein C_ fast-type	2080	0.74
Q5SX39	Myosin-4	19417	0.73
Q03265	ATP synthase subunit alpha_ mitochondrial	667	0.73
P31001	Desmin	117	0.73
P14152	Malate dehydrogenase_ cytoplasmic	409	0.73
P08249	Malate dehydrogenase_ mitochondrial O	1446	0.73
Q9D0F9	Phosphoglucomutase-1	201	0.73
P58774	Tropomyosin beta chain	7827	0.72
Q02566	Myosin-6	5984	0.71
P13542	Myosin-8	11378	0.71
P15532	Nucleoside diphosphate kinase A	281	0.71
P57780	Alpha-actinin-4	178	0.70
Q5SX40	Myosin-1	14904	0.70
Q91Z83	Myosin-7	7429	0.70
P07724	Serum albumin	1403	0.70
P17182	Alpha-enolase	4627	0.69
Q9JKS4	LIM domain-binding protein 3	850	0.69
Q60605	Myosin light polypeptide 6	4175	0.69
P09041	Phosphoglycerate kinase 2	639	0.69
P09542	Myosin light chain 3	9702	0.68
Q64518	Sarcoplasmic/endoplasmic reticulum calcium ATPase 3	130	0.68
P13541	Myosin-3	4714	0.68
P05201	Aspartate aminotransferase_ cytoplasmic	85	0.67
P16015	Carbonic anhydrase 3	2095	0.67
P06151	L-lactate dehydrogenase A chain	2596	0.67
P20801	Troponin C_ skeletal muscle	1409	0.67
Q6P8J7	Creatine kinase S-type_ mitochondrial	389	0.66
P09411	Phosphoglycerate kinase 1	686	0.66

O88990	Alpha-actinin-3	2430	0.65
P00342	L-lactate dehydrogenase C chain	1187	0.65
Q9JI91	Alpha-actinin-2	1054	0.63
Q7TPR4	Alpha-actinin-1	193	0.63
P56480	ATP synthase subunit beta_ mitochondrial	1129	0.63
P0DP26	Calmodulin-1	502	0.63
O70250	Phosphoglycerate mutase 2	1961	0.63
P0DP28	Calmodulin-3	502	0.62
P21550	Beta-enolase	9402	0.61
P0DP27	Calmodulin-2	502	0.61
P17183	Gamma-enolase	444	0.61
P05064	Fructose-bisphosphate aldolase A	18392	0.61
P17751	Triosephosphate isomerase	6732	0.61
P16125	L-lactate dehydrogenase B chain	353	0.60
Q64478	Histone H2B type 1-H	890	0.59
P68134	Actin_ alpha skeletal muscle	111877	0.59
P10126	Elongation factor 1-alpha 1	108	0.59
P04247	Myoglobin	304	0.59
Q8BFZ3	Beta-actin-like protein 2	26045	0.58
Q64475	Histone H2B type 1-B	890	0.58
P10853	Histone H2B type 1-F/J/L	890	0.58
Q64525	Histone H2B type 2-B	890	0.58
Q9QXS1	Plectin	100	0.58
Q6ZWY9	Histone H2B type 1-C/E/G	890	0.58
Q8CGP1	Histone H2B type 1-K	890	0.58
P68033	Actin_ alpha cardiac muscle 1	106303	0.57
P05063	Fructose-bisphosphate aldolase C	1869	0.57
P70696	Histone H2B type 1-A	708	0.57
Q9D2U9	Histone H2B type 3-A	890	0.57
Q8CGP0	Histone H2B type 3-B	890	0.57
Q9JK37	Myozenin-1	466	0.57
P63268	Actin_ gamma-enteric smooth muscle	102366	0.57
P62631	Elongation factor 1-alpha 2	417	0.57
Q8CGP2	Histone H2B type 1-P	890	0.57
P62737	Actin_ aortic smooth muscle	102579	0.56
Q64524	Histone H2B type 2-E	890	0.55
P09541	Myosin light chain 4	6543	0.55
P10854	Histone H2B type 1-M	890	0.54
Q9JIF9	Myotilin	216	0.52
Q9D051	Pyruvate dehydrogenase E1 component subunit beta_ mitochondrial	88	0.51
Q3UU96	Serine/threonine-protein kinase MRCK alpha	128	0.50
P05977	Myosin light chain 1/3 skeletal muscle isoform	33434	0.50
P16858	Glyceraldehyde-3-phosphate dehydrogenase	25084	0.48
P01942	Hemoglobin subunit alpha	10325	0.45
P02104	Hemoglobin subunit epsilon-Y2	2425	0.44
Q99PT9	Kinesin-like protein KIF19	181	0.41
P02088	Hemoglobin subunit beta-1	3958	0.41
Q8CB87	Ras-related protein Rab-44	115	0.37
P99024	Tubulin beta-5 chain	222	0.34

Q9D6F9	Tubulin beta-4A chain	1641	0.32
Q9WVP9	Interferon-induced GTP-binding protein Mx2	298	0.32
P68372	Tubulin beta-4B chain	161	0.31
Q7TMM9	Tubulin beta-2A chain	222	0.31
Q9CWF2	Tubulin beta-2B chain	222	0.31
Q3U435	Matrix metalloproteinase-25	93	0.30
P09922	Interferon-induced GTP-binding protein Mx1	327	0.30
Q61503	5'-nucleotidase	105	0.29
P32848	Parvalbumin alpha	7739	0.28
Q8VHX6	Filamin-C	118	0.23
Q3UVV9	von Willebrand factor A domain-containing protein 3A	118	0.12
Q8VI93	2'-5'-oligoadenylate synthase 3	51	SI*
P20029	78 kDa glucose-regulated protein	46	SI
Q5SWU9	Acetyl-CoA carboxylase 1	123	SI
Q9QY83	Actin-like protein 7B	95	SI
P03958	Adenosine deaminase	71	SI
Q8C0T9	Adenylate cyclase type 10	30	SI
P46660	Alpha-internexin	72	SI
G3UZ78	Androglobin	97	SI
Q9WV74	Ankyrin repeat and SOCS box protein 1	243	SI
O88512	AP-1 complex subunit gamma-like 2	58	SI
P12382	ATP-dependent 6-phosphofructokinase liver type	77	SI
Q9WUA3	ATP-dependent 6-phosphofructokinase_ platelet type	85	SI
Q8C0J2	Autophagy-related protein 16-1	46	SI
O70318	Band 4.1-like protein 2	31	SI
P21855	B-cell differentiation antigen CD72	88	SI
Q91Z96	BMP-2-inducible protein kinase	100	SI
Q8VHF2	Cadherin-related family member 5	31	SI
Q80V31	Centrosomal protein of 104 kDa	237	SI
Q8C6E0	Cilia- and flagella-associated protein 36	171	SI
Q9D180	Cilia- and flagella-associated protein 57	51	SI
E9Q1U1	Coiled-coil domain-containing protein 171	74	SI
P11087	Collagen alpha-1(I) chain	70	SI
P02463	Collagen alpha-1(IV) chain	96	SI
Q07643	Collagen alpha-2(IX) chain	63	SI
Q04447	Creatine kinase B-type	52	SI
O88874	Cyclin-K	91	SI
P00405	Cytochrome c oxidase subunit 2	97	SI
P43023	Cytochrome c oxidase subunit 6A2_ mitochondrial	1471	SI
Q8R1A4	Dedicator of cytokinesis protein 7	109	SI
A2RSQ0	DENN domain-containing protein 5B	79	SI
O08749	Dihydrolipoyl dehydrogenase_ mitochondrial	81	SI
Q9EQF6	Dihydropyrimidinase-related protein 5	120	SI
Q9DA79	Dipeptidase 3	80	SI
Q811D0	Disks large homolog 1	63	SI
P22682	E3 ubiquitin-protein ligase CBL	60	SI
Q4U2R1	E3 ubiquitin-protein ligase HERC2	226	SI
Q8R0K2	E3 ubiquitin-protein ligase TRIM31	58	SI
Q99MI1	ELKS/Rab6-interacting/CAST family member1	65	SI

Q62420	Endophilin-A1	71	SI
Q62419	Endophilin-A2	71	SI
P48299	Endothelin-3	139	SI
Q9ERK4	Exportin-2	93	SI
Q5RJI4	Extracellular tyrosine-protein kinase PKDCC	290	SI
A3KGK3	Fer-1-like protein 4	107	SI
Q9JJN1	Fibroblast growth factor 21	137	SI
Q8BY35	FYVE_RhoGEF and PH domain-containing protein 2	55	SI
Q9DBA9	General transcription factor IIH subunit 1	82	SI
Q00612	Glucose-6-phosphate 1-dehydrogenase X	95	SI
Q9WU65	Glycerol kinase 2	53	SI
Q9JLM9	Growth factor receptor-bound protein 14	120	SI
Q9ERL9	Guanylate cyclase soluble subunit alpha-3	67	SI
P79457	Histone demethylase UTY	42	SI
Q9Z148	Histone-lysine N-methyltransferase EHMT2	84	SI
Q63ZW7	InaD-like protein	41	SI
Q8CIM8	Integrator complex subunit 4	86	SI
A2ARA8	Integrin alpha-8	46	SI
Q80U22	Iporin	131	SI
Q69ZK5	Kelch-like protein 14	91	SI
Q9D312	Keratin_ type I cytoskeletal 20	60	SI
L0N7N1	Kinesin-like protein KIF14	105	SI
Q99PW8	Kinesin-like protein KIF17	51	SI
Q80WE4	Kinesin-like protein KIF20B	49	SI
E9Q5G3	Kinesin-like protein KIF23	47	SI
Q9D3W5	Leucine-rich repeat-containing protein 71	122	SI
Q8CBY3	Leukocyte receptor cluster member 8 homolog	69	SI
Q148V7	LisH domain and HEAT repeat-containing protein KIAA1468	103	SI
Q61790	Lymphocyte activation gene 3 protein	90	SI
Q8CDB0	MAP kinase-interacting serine/threonine- protein kinase 2	147	SI
Q8VCD5	Mediator of RNA polymerase II transcription subunit 17	68	SI
O35954	Membrane-associated phosphatidylinositol transfer protein 1	86	SI
Q3TPJ7	Midnolin	89	SI
Q99MT2	MutS protein homolog 4	76	SI
Q80YT7	Myomegalin	78	SI
Q3UIZ8	Myosin light chain kinase 3	138	SI
Q91XS1	Myotubularin-related protein 4	153	SI
Q8R4E4	Myozenin-3	277	SI
Q66X22	NACHT_LRR and PYD domains-containing protein 9B	120	SI
Q99LC3	NADH dehydrogenase [ubiquinone] 1 alpha subcomplex subunit 10_mitochondrial	149	SI
Q91YT0	NADH dehydrogenase [ubiquinone] flavoprotein 1_ mitochondrial	76	SI
Q91WD5	NADH dehydrogenase [ubiquinone] iron- sulfur protein 2_ mitochondrial	89	SI
E9Q7X7	Neurexin II	64	SI
Q6ZQ12	Ninein-like protein	58	SI
Q69ZF3	Non-lysosomal glucosylceramidase	66	SI
Q99ML2	Non-receptor tyrosine-protein kinase TNK1	50	SI
Q9WU42	Nuclear receptor corepressor 2	25	SI
P41593	Parathyroid hormone/parathyroid hormone- related peptide receptor	65	SI

Q8CEE6	PAS domain-containing serine/threonine- protein kinase	63	SI
P15331	Peripherin	83	SI
Q78Y63	Phosducin-like protein 2	95	SI
Q9DBJ1	Phosphoglycerate mutase 1	30	SI
Q3UH93	Plexin-D1	149	SI
Q62083	PRKCA-binding protein	81	SI
P54823	Probable ATP-dependent RNA helicase DDX6	67	SI
Q8CDU6	Probable E3 ubiquitin-protein ligase HECTD2	359	SI
Q8VDC0	Probable leucine--tRNA ligase_ mitochondrial	93	SI
Q9QUM9	Proteasome subunit alpha type-6	86	SI
O70279	Protein DGCR14	68	SI
Q9D4K5	Protein FAM166A	58	SI
Q2VWQ2	Protein kinase C-binding protein NELL1	117	SI
B1AUR6	Protein MMS22-like	162	SI
Q8K2C7	Protein OS-9	76	SI
P97352	Protein S100-A13	135	SI
Q80TF3	Protocadherin-19	48	SI
Q60695	Ral guanine nucleotide dissociation stimulator- like 1	65	SI
Q91YQ1	Ras-related protein Rab-7L1	92	SI
A2AQ19	RNA polymerase-associated protein RTF1 homolog	64	SI
Q60806	Serine/threonine-protein kinase PLK3	77	SI
Q8BKX6	Serine/threonine-protein kinase SMG1	100	SI
Q9Z2E3	Serine/threonine-protein kinase/endoribonuclease IRE2	64	SI
Q9JID9	SH2B adapter protein 2	218	SI
Q8BJA2	Solute carrier family 41 member 1	134	SI
Q3UTJ2	Sorbin and SH3 domain-containing protein 2	136	SI
Q8BI29	Specifically androgen-regulated gene protein	170	SI
Q7TME2	Sperm-associated antigen 5	51	SI
Q5U4C3	Splicing factor_ arginine/serine-rich 19	208	SI
Q9QWI6	SRC kinase signaling inhibitor 1	41	SI
Q91YE8	Synaptopodin-2	152	SI
P70327	T-box transcription factor TBX6	72	SI
A3KMP2	Tetratricopeptide repeat protein 38	69	SI
Q715T0	Thioredoxin domain-containing protein 3	136	SI
Q8K424	Transient receptor potential cation channel subfamily V member 3	59	SI
Q8BGN6	Transmembrane gamma-carboxyglutamic acid protein 4	124	SI
Q922K9	Tyrosine-protein kinase FRK	61	SI
Q9D0L4	Uncharacterized aarF domain-containing protein kinase 1	86	SI
Q3TEI4	Uncharacterized protein C15orf39 homolog	120	SI
Q3TLD5	Unconventional prefoldin RPB5 interactor	81	SI
Q91ZJ5	UTP--glucose-1-phosphate uridylyltransferase	73	SI
Q5KU39	Vacuolar protein sorting-associated protein 41 homolog	49	SI
P29533	Vascular cell adhesion protein 1	84	SI
Q8VDJ3	Vigilin	383	SI
Q60932	Voltage-dependent anion-selective channel protein 1	291	SI
Q3U5F4	YrdC domain-containing protein_ mitochondrial	103	SI
Q5F293	Zinc finger and BTB domain-containing protein 4	71	SI
Q9JJN2	Zinc finger homeobox protein 4	199	SI
Q9JLM4	Zinc finger MYM-type protein 3	76	SI
Q8CJ19	[F-actin]-methionine sulfoxide oxidase MICAL3	80	RI
Q8K4S1	1-phosphatidylinositol 4_5-bisphosphate phosphodiesterase epsilon-1	225	RI
Q9EQC1	3 beta-hydroxysteroid dehydrogenase type 7	491	RI

P97819	85/88 kDa calcium-independent phospholipase A2	373	RI
Q8QZT1	Acetyl-CoA acetyltransferase_ mitochondrial	146	RI
Q80WC9	Acyl-CoA synthetase family member 4	55	RI
P48962	ADP/ATP translocase 1	48	RI
P51881	ADP/ATP translocase 2	48	RI
Q3V132	ADP/ATP translocase 4	50	RI
P07758	Alpha-1-antitrypsin 1-1	132	RI
P22599	Alpha-1-antitrypsin 1-2	132	RI
Q00896	Alpha-1-antitrypsin 1-3	132	RI
Q00897	Alpha-1-antitrypsin 1-4	132	RI
Q9QYC0	Alpha-adducin	130	RI
Q8CFA2	Aminomethyltransferase_ mitochondrial	140	RI
Q80VM7	Ankyrin repeat domain-containing protein 24	250	RI
Q3UMR0	Ankyrin repeat domain-containing protein 27	212	RI
Q8C8R3	Ankyrin-2	131	RI
P14824	Annexin A6	111	RI
Q9JKC8	AP-3 complex subunit mu-1	76	RI
Q80V94	AP-4 complex subunit epsilon-1	38	RI
Q5YD48	APOBEC1 complementation factor	92	RI
Q9Z2A5	Arginyl-tRNA--protein transferase 1	113	RI
Q8R4I1	Ataxin-7	99	RI
Q9CQQ7	ATP synthase F(0) complex subunit B1_ mitochondrial	193	RI
Q8VDW0	ATP-dependent RNA helicase DDX39A	161	RI
Q60936	Atypical kinase COQ8A_ mitochondrial	211	RI
P52963	Band 4.1-like protein 4A	100	RI
P48754	Breast cancer type 1 susceptibility protein homolog	160	RI
Q9WV35	C->U-editing enzyme APOBEC-2	386	RI
Q9WTR5	Cadherin-13	77	RI
Q6Q473	Calcium-activated chloride channel regulator4A	84	RI
Q9D6P8	Calmodulin-like protein 3	117	RI
Q3UKK2	Carcinoembryonic antigen-related cell adhesion molecule 5	54	RI
Q61301	Catenin alpha-2	67	RI
O54724	Caveolae-associated protein 1	127	RI
Q14B71	Cell division cycle-associated protein 2	464	RI
Q6P8Y0	Cilia- and flagella-associated protein 161	101	RI
Q9CZU6	Citrate synthase_ mitochondrial	121	RI
P49025	Citron Rho-interacting kinase	510	RI
Q68FD5	Clathrin heavy chain 1	91	RI
O35218	Cleavage and polyadenylation specificity factor subunit 2	81	RI
Q640L5	Coiled-coil domain-containing protein 18	63	RI
Q6NS45	Coiled-coil domain-containing protein 66	107	RI
Q504P2	C-type lectin domain family 12 member A	115	RI
Q99KY4	Cyclin-G-associated kinase	199	RI
P12787	Cytochrome c oxidase subunit 5A_ mitochondrial	539	RI
P62897	Cytochrome c_somatic	412	RI
Q570Y9	DEP domain-containing mTOR-interacting protein	133	RI
Q8CDG3	Deubiquitinating protein VCIP135	57	RI
P70175	Disks large homolog 3	122	RI
Q8R4E9	DNA replication factor Cdt1	132	RI
Q99LC5	Electron transfer flavoprotein subunit alpha_ mitochondrial	122	RI
P58252	Elongation factor 2	125	RI
Q8K203	Endonuclease 8-like 3	161	RI
P42567	Epidermal growth factor receptor substrate 15	58	RI

Q8C7X2	ER membrane protein complex subunit 1	77	RI
P47753	F-actin-capping protein subunit alpha-1	90	RI
P47754	F-actin-capping protein subunit alpha-2	289	RI
P50608	Fibromodulin	68	RI
Q80TD3	Folliculin-interacting protein 2	39	RI
Q99JB6	Forkhead box protein P3	70	RI
Q8VDC1	FYVE and coiled-coil domain-containing protein 1	72	RI
Q60928	Gamma-glutamyltranspeptidase 1	219	RI
Q9ESZ8	General transcription factor II-I	141	RI
Q99NI3	General transcription factor II-I repeat domain-containing protein 2	182	RI
Q9CQI3	Glia maturation factor beta	283	RI
Q64467	Glyceraldehyde-3-phosphate dehydrogenase_ testis-specific	108	RI
Q8BKV1	Glypican-2	69	RI
Q60780	Growth arrest-specific protein 7	110	RI
Q60779	Growth arrest-specific protein 8	52	RI
P11499	Heat shock protein HSP 90-beta	105	RI
Q99NG0	Helicase ARIP4	77	RI
A2AJ76	Hemicentin-2	204	RI
Q8BRB7	Histone acetyltransferase KAT6B	193	RI
P97443	Histone-lysine N-methyltransferase Smyd1	79	RI
Q9WUI0	Homeobox protein MIXL1	152	RI
P01586	Interleukin-3	140	RI
P54071	Isocitrate dehydrogenase [NADP]_ mitochondrial O	166	RI
Q497I4	Keratin_ type I cuticular Ha5	139	RI
Q61595	Kinectin	135	RI
Q9QXL1	Kinesin-like protein KIF21B	77	RI
O89112	LanC-like protein 1	101	RI
Q8CGA3	Large neutral amino acids transporter small subunit 4	92	RI
Q8C129	Leucyl-cystinyl aminopeptidase	88	RI
Q9DBN5	Lon protease homolog 2_peroxisomal	80	RI
P34884	Macrophage migration inhibitory factor	163	RI
Q9WV34	MAGUK p55 subfamily member 2	165	RI
Q924M7	Mannose-6-phosphate isomerase	111	RI
Q9JI70	McKusick-Kaufman/Bardet-Biedl syndromes putative chaperonin	99	RI
Q5F2C3	Meiosis-specific kinetochore protein	87	RI
B1AYB6	Methyl-CpG-binding domain protein 5	114	RI
Q9DCS2	Methyltransferase-like 26	319	RI
Q8CAQ8	MICOS complex subunit Mic60	244	RI
Q8C052	Microtubule-associated protein 1S	82	RI
Q9WTX8	Mitotic spindle assembly checkpoint protein MAD1	51	RI
P30306	M-phase inducer phosphatase 2	109	RI
P23949	mRNA decay activator protein ZFP36L2	65	RI
O08539	Myc box-dependent-interacting protein 1	200	RI
Q3UIJ9	Myocardial zonula adherens protein	48	RI
Q8CI43	Myosin light chain 6B	175	RI
P70402	Myosin-binding protein H	207	RI
Q923E4	NAD-dependent protein deacetylase sirtuin-1	170	RI
Q8BMT4	Negative regulator of reactive oxygen species	382	RI
P70232	Neural cell adhesion molecule L1-like protein	174	RI
Q99PJ0	Neurotrimin	122	RI
P70255	Nuclear factor 1 C-type	196	RI
P25799	Nuclear factor NF-kappa-B p105 subunit	172	RI
Q99MH5	Nucleoside diphosphate kinase homolog 5	235	RI

O70209	PDZ and LIM domain protein 3	103	RI
P17742	Peptidyl-prolyl cis-trans isomerase A	501	RI
P30416	Peptidyl-prolyl cis-trans isomerase FKBP4	75	RI
P48725	Pericentrin	138	RI
Q62009	Periostin	90	RI
P16331	Phenylalanine-4-hydroxylase	131	RI
Q69ZK0	Phosphatidylinositol 3_4_5-trisphosphate- dependent Rac exchanger 1 protein	44	RI
Q61194	Phosphatidylinositol 4-phosphate 3-kinase C2 domain-containing subunit alpha	49	RI
P70181	Phosphatidylinositol 4-phosphate 5-kinase type-1 beta	64	RI
Q9Z280	Phospholipase D1	151	RI
B2RPU2	Pleckstrin homology domain-containing family D member 1	87	RI
P01193	Pro-opiomelanocortin	84	RI
Q8C569	Protein FAM118B	197	RI
Q148A4	Protein phosphatase 1 regulatory subunit 32	156	RI
Q9Z1N9	Protein unc-13 homolog B	87	RI
O55134	Protocadherin-12	196	RI
Q7TSK3	Protocadherin-8	68	RI
P06240	Proto-oncogene tyrosine-protein kinase LCK	1024	RI
Q8BRM2	RAB6-interacting golgin	184	RI
P59729	Ras and Rab interactor 3	129	RI
Q8CGE9	Regulator of G-protein signaling 12	59	RI
Q9DC04	Regulator of G-protein signaling 3	91	RI
P00796	Renin-2	110	RI
Q811M1	Rho GTPase-activating protein 15	83	RI
Q9CWR0	Rho guanine nucleotide exchange factor 25	129	RI
O35130	Ribosomal RNA small subunit methyltransferase NEP1	228	RI
Q4VGL6	Roquin-1	143	RI
E9PZQ0	Ryanodine receptor 1	131	RI
Q60988	SCL-interrupting locus protein homolog	104	RI
Q64105	Sepiapterin reductase	148	RI
Q9JIY5	Serine protease HTRA2_ mitochondrial	191	RI
Q7TSI3	Serine/threonine-protein phosphatase 6 regulatory subunit 1	141	RI
Q8BTK5	SET and MYND domain-containing protein 4	64	RI
Q4ACU6	SH3 and multiple ankyrin repeat domains protein 3	163	RI
P98083	SHC-transforming protein 1	71	RI
Q8VDU5	SNF-related serine/threonine-protein kinase	57	RI
Q9D2S4	Sperm acrosome-associated protein 7	218	RI
Q80ZX8	Sperm-associated antigen 1	93	RI
Q9Z1N5	Spliceosome RNA helicase Ddx39b	81	RI
P97496	SWI/SNF complex subunit SMARCC1	96	RI
Q8BWB1	Synaptopodin 2-like protein	170	RI
Q8K1E0	Syntaxin-5	496	RI
P26039	Talin-1	147	RI
Q8CGA2	TBC1 domain family member 14	168	RI
Q6X6Z7	Tektin-3	78	RI
O70548	Telethonin	132	RI
Q9JMH6	Thioredoxin reductase 1_ cytoplasmic	114	RI
Q9JLT4	Thioredoxin reductase 2_ mitochondrial	234	RI
P63058	Thyroid hormone receptor alpha	137	RI
Q60610	T-lymphoma invasion and metastasis-inducing protein 1	57	RI
Q8BHE4	Transmembrane protein 108	70	RI

O70472	Transmembrane protein 131	134	RI
Q64514	Tripeptidyl-peptidase 2	96	RI
Q9WUZ5	Troponin I_ slow skeletal muscle	233	RI
Q9ERD7	Tubulin beta-3 chain	228	RI
Q922F4	Tubulin beta-6 chain	157	RI
A4Q9F4	Tubulin polyglutamylase TTLL11	103	RI
Q8BX43	Tumor necrosis factor receptor superfamily member 19L	147	RI
Q9Z315	U4/U6.U5 tri-snRNP-associated protein 1	79	RI
P56399	Ubiquitin carboxyl-terminal hydrolase 5	119	RI
Q8CB27	Ubiquitin thioesterase OTU1	137	RI
P68037	Ubiquitin-conjugating enzyme E2 L3	79	RI
Q3UTZ3	Uncharacterized protein C7orf43 homolog	87	RI
A9Z1V5	von Willebrand factor A domain-containing protein 5B1	171	RI
Q6PDJ1	VWFA and cache domain-containing protein 1	53	RI
Q80VW5	Whirlin	184	RI
Q6NZF1	Zinc finger CCCH domain-containing protein11A	107	RI
Q8BYK8	Zinc finger CCCH domain-containing protein6	114	RI
Q9R0G7	Zinc finger E-box-binding homeobox 2	178	RI
Q8JZL0	Zinc finger protein 467	222	RI
Q8K083	Zinc finger protein 536	412	RI

[a]Identification is based on proteins ID from UniProt protein database, reviewed only (http://www.uniprot.org/).
[b]Proteins with expression significantly altered are organized according to the ratio.
*Indicates unique proteins in alphabetical order.

Table S5. Proteins with expression significantly altered in the gastrocnemius of SII (A/J, water containing 50 ppm F, no-exercise) and RII (129P3/J, water containing 50 ppm F, no-exercise) mice

[a]**Acession number**	**Protein name**	**PLGS Score**	[b]***Ratio* SII:RII**
P32848	Parvalbumin alpha	1413	18.36
Q64467	Glyceraldehyde-3-phosphate dehydrogenase testis-specific	204	6.82
Q91VW5	Golgin subfamily A member 4	84	4.06
P21107	Tropomyosin alpha-3 chain	148	3.82
P11404	Fatty acid-binding protein_ heart	393	3.42
Q04690	Neurofibromin	60	2.61
P51881	ADP/ATP translocase 2	217	2.51
O09165	Calsequestrin-1	1611	2.51
P62897	Cytochrome c_ somatic	145	2.46
P48962	ADP/ATP translocase 1	257	2.39
A2AQP0	Myosin-7B	1485	2.34
P13412	Troponin I_ fast skeletal muscle	2483	2.32
Q3V1D3	AMP deaminase 1	175	2.18
P57780	Alpha-actinin-4	172	2.05
P02104	Hemoglobin subunit epsilon-Y2	2086	2.05
Q9QXS1	Plectin	28	1.92
P02089	Hemoglobin subunit beta-2	2086	1.90
P52480	Pyruvate kinase PKM	5611	1.88
O55143	Sarcoplasmic/endoplasmic reticulum calcium ATPase 2	205	1.82
P12382	ATP-dependent 6-phosphofructokinase_liver type	68	1.77
O70250	Phosphoglycerate mutase 2	1811	1.77
P02088	Hemoglobin subunit beta-1	2777	1.73
Q9R0Y5	Adenylate kinase isoenzyme 1	7048	1.68

Q7TQ48	Sarcalumenin	60	1.68
P0DP28	Calmodulin-3	315	1.67
Q9JI91	Alpha-actinin-2	377	1.65
P0DP26	Calmodulin-1	315	1.65
P17751	Triosephosphate isomerase	4428	1.65
Q7TPR4	Alpha-actinin-1	215	1.63
P0DP27	Calmodulin-2	315	1.62
Q3TJD7	PDZ and LIM domain protein 7	950	1.62
P08249	Malate dehydrogenase_ mitochondrial	756	1.60
A2AL36	Centriolin	88	1.57
P16858	Glyceraldehyde-3-phosphate dehydrogenase	19243	1.57
P07310	Creatine kinase M-type	17477	1.52
Q8R429	Sarcoplasmic/endoplasmic reticulum calcium ATPase 1	753	1.52
P05201	Aspartate aminotransferase_ cytoplasmic	393	1.49
P17183	Gamma-enolase	799	1.49
Q9CR68	Cytochrome b-c1 complex subunit Rieske_ mitochondrial	86	1.48
Q5SSE9	ATP-binding cassette sub-family A member13	128	1.46
P14152	Malate dehydrogenase_ cytoplasmic	150	1.46
Q99LX0	Protein DJ-1	956	1.45
P12787	Cytochrome c oxidase subunit 5A_ mitochondrial	1033	1.43
P10126	Elongation factor 1-alpha 1	82	1.43
P06151	L-lactate dehydrogenase A chain	2878	1.43
P47857	ATP-dependent 6- phosphofructokinase_muscle type	444	1.39
P31001	Desmin	185	1.39
O88990	Alpha-actinin-3	1519	1.38
Q9D0F9	Phosphoglucomutase-1	153	1.38
P07724	Serum albumin	345	1.36
P60710	Actin_ cytoplasmic 1	94968	1.35
P63260	Actin_ cytoplasmic 2	94968	1.35
P04247	Myoglobin	434	1.35
Q01768	Nucleoside diphosphate kinase B	141	1.35
P13542	Myosin-8	10380	1.34
P15532	Nucleoside diphosphate kinase A	141	1.34
P58771	Tropomyosin alpha-1 chain	12103	1.34
Q5SX40	Myosin-1	13724	1.32
Q99KI0	Aconitate hydratase_ mitochondrial	145	1.31
Q5XKE0	Myosin-binding protein C_ fast-type	1808	1.31
P53657	Pyruvate kinase PKLR	698	1.31
P05202	Aspartate aminotransferase_ mitochondrial	117	1.30
P63017	Heat shock cognate 71 kDa protein	117	1.30
Q62234	Myomesin-1	143	1.30
P13541	Myosin-3	4303	1.30
P09411	Phosphoglycerate kinase 1	1372	1.30
P09041	Phosphoglycerate kinase 2	992	1.30
Q03265	ATP synthase subunit alpha_ mitochondrial	549	1.28
P62737	Actin_ aortic smooth muscle	110654	1.26
P63268	Actin_ gamma-enteric smooth muscle	110480	1.26
P56480	ATP synthase subunit beta_ mitochondrial	791	1.26
P16125	L-lactate dehydrogenase B chain	225	1.26
P00342	L-lactate dehydrogenase C chain	1353	1.26
P97457	Myosin regulatory light chain 2_ skeletal muscle isoform	13666	1.26
P68033	Actin_ alpha cardiac muscle 1	117501	1.25
P68134	Actin_ alpha skeletal muscle	121903	1.25

P17156	Heat shock-related 70 kDa protein 2	98	1.25
P05064	Fructose-bisphosphate aldolase A	17992	1.23
Q91Z83	Myosin-7	7181	1.23
P21550	Beta-enolase	6260	1.22
Q02566	Myosin-6	5961	1.22
P16015	Carbonic anhydrase 3	2304	1.21
P09542	Myosin light chain 3	3753	1.21
P20801	Troponin C_ skeletal muscle	677	1.21
P58774	Tropomyosin beta chain	6894	1.20
Q61879	Myosin-10	166	1.17
O08638	Myosin-11	165	1.16
P62631	Elongation factor 1-alpha 2	208	1.15
Q9JKS4	LIM domain-binding protein 3	690	1.15
Q60605	Myosin light polypeptide 6	1250	1.15
Q8VDD5	Myosin-9	165	1.14
P17182	Alpha-enolase	3491	1.13
Q8BFZ3	Beta-actin-like protein 2	19443	1.13
Q6URW6	Myosin-14	279	1.13
Q9QZ47	Troponin T_ fast skeletal muscle	1994	1.12
P05977	Myosin light chain 1/3_ skeletal muscle isoform	24671	1.11
P09541	Myosin light chain 4	4125	1.11
Q5SX39	Myosin-4	19719	1.11
Q9WUB3	Glycogen phosphorylase_ muscle form	5410	1.05
P05063	Fructose-bisphosphate aldolase C	2425	0.87
P42128	Forkhead box protein K1	112	0.61
Q8CHI8	E1A-binding protein p400	77	0.34
Q3UH93	Plexin-D1	206	0.19
Q7TMW6	Cytosolic Fe-S cluster assembly factor NARFL	147	0.15
Q810T2	G2/mitotic-specific cyclin-B3	61	0.12
P70327	T-box transcription factor TBX6	59	0.12
Q4KUS2	Protein unc-13 homolog A	193	0.11
O08810	116 kDa U5 small nuclear ribonucleoprotein component	394	SII*
P62259	14-3-3 protein epsilon	107	SII
Q8VCR2	17-beta-hydroxysteroid dehydrogenase 13	124	SII
P20029	78 kDa glucose-regulated protein	171	SII
P54822	Adenylosuccinate lyase	90	SII
G3X982	Aldehyde oxidase 3	32	SII
P53995	Anaphase-promoting complex subunit 1	68	SII
Q8BZQ7	Anaphase-promoting complex subunit 2	63	SII
Q9D4H4	Angiomotin-like protein 1	50	SII
Q9WVH6	Angiopoietin-4	154	SII
Q99NH0	Ankyrin repeat domain-containing protein 17	94	SII
O88879	Apoptotic protease-activating factor 1	69	SII
A2RTL5	Arginine/serine-rich coiled-coil protein 2	344	SII
Q8BIP0	Aspartate--tRNA ligase_ mitochondrial	41	SII
Q4QY64	ATPase family AAA domain-containing protein 5	30	SII
Q9DC29	ATP-binding cassette sub-family B member6_ mitochondrial	69	SII
Q9WUA3	ATP-dependent 6-phosphofructokinase_ platelet type	33	SII
O88738	Baculoviral IAP repeat-containing protein 6	80	SII
P21855	B-cell differentiation antigen CD72	68	SII
P41183	B-cell lymphoma 6 protein homolog	80	SII
Q6PAL0	BEN domain-containing protein 3	33	SII
Q8BWG8	Beta-arrestin-1	526	SII

Q9WV35	C->U-editing enzyme APOBEC-2	578	SII
B9EHT4	CAP-Gly domain-containing linker protein 3	265	SII
A2A6Q5	Cell division cycle protein 27 homolog	61	SII
Q6RT24	Centromere-associated protein E	95	SII
D2J0Y4	Centrosomal protein C10orf90 homolog	230	SII
Q80TV8	CLIP-associating protein 1	51	SII
E9Q1U1	Coiled-coil domain-containing protein 171	57	SII
Q6PHN1	Coiled-coil domain-containing protein 57	60	SII
Q6NS45	Coiled-coil domain-containing protein 66	66	SII
Q04447	Creatine kinase B-type	70	SII
P30275	Creatine kinase U-type_ mitochondrial	180	SII
Q9DB77	Cytochrome b-c1 complex subunit 2_ mitochondrial	135	SII
Q91YE9	Cytosolic 5'-nucleotidase 1B	88	SII
Q09M02	Cytosolic carboxypeptidase-like protein 5	142	SII
Q8BIK4	Dedicator of cytokinesis protein 9	51	SII
A6H8H2	DENN domain-containing protein 4C	29	SII
Q7TMD7	Desmoglein-4	58	SII
O08749	Dihydrolipoyl dehydrogenase_ mitochondrial	39	SII
Q8BMF4	Dihydrolipoyllysine-residue acetyltransferase component of pyruvate dehydrogenase complex_ mitochondrial	115	SII
Q9DBT9	Dimethylglycine dehydrogenase_ mitochondrial	65	SII
Q811D0	Disks large homolog 1	107	SII
Q91XM9	Disks large homolog 2	50	SII
Q9JMC3	DnaJ homolog subfamily A member 4	166	SII
O70469	Docking protein 2	75	SII
Q4U2R1	E3 ubiquitin-protein ligase HERC2	277	SII
Q8C669	E3 ubiquitin-protein ligase pellino homolog 1	57	SII
A2AN08	E3 ubiquitin-protein ligase UBR4	72	SII
Q9DCW4	Electron transfer flavoprotein subunit beta	151	SII
Q99MI1	ELKS/Rab6-interacting/CAST family member1	49	SII
P58252	Elongation factor 2	430	SII
Q8C0D5	Elongation factor-like GTPase 1	97	SII
Q9ERK4	Exportin-2	86	SII
Q3TR08	Fibronectin type III domain-containing protein4	119	SII
Q8VHX6	Filamin-C	40	SII
P58462	Forkhead box protein P1	155	SII
Q99JB6	Forkhead box protein P3	100	SII
Q8VDC1	FYVE and coiled-coil domain-containing protein 1	46	SII
O88741	Ganglioside-induced differentiation-associated protein 1	96	SII
Q9Z1Z0	General vesicular transport factor p115	46	SII
Q5SNZ0	Girdin	86	SII
P19157	Glutathione S-transferase P 1	114	SII
P46425	Glutathione S-transferase P 2	95	SII
P13707	Glycerol-3-phosphate dehydrogenase[NAD(+)]_ cytoplasmic	73	SII
P36916	Guanine nucleotide-binding protein-like 1	78	SII
O54865	Guanylate cyclase soluble subunit beta-1	74	SII
Q5PRF0	HEAT repeat-containing protein 5A	106	SII
P11499	Heat shock protein HSP 90-beta	47	SII
P59438	Hermansky-Pudlak syndrome 5 protein homolog	107	SII
P70696	Histone H2B type 1-A	1016	SII
Q64475	Histone H2B type 1-B	1132	SII
Q6ZWY9	Histone H2B type 1-C/E/G	1132	SII
P10853	Histone H2B type 1-F/J/L	1132	SII

Q64478	Histone H2B type 1-H	1132	SII
Q8CGP1	Histone H2B type 1-K	1132	SII
P10854	Histone H2B type 1-M	1132	SII
Q8CGP2	Histone H2B type 1-P	1132	SII
Q64525	Histone H2B type 2-B	1132	SII
Q64524	Histone H2B type 2-E	1132	SII
Q9D2U9	Histone H2B type 3-A	1132	SII
Q8CGP0	Histone H2B type 3-B	1132	SII
P97443	Histone-lysine N-methyltransferase Smyd1	66	SII
O08934	Homeobox protein unc-4 homolog	120	SII
O35344	Importin subunit alpha-4	58	SII
Q3USB7	Inactive phospholipase C-like protein 1	412	SII
O88351	Inhibitor of nuclear factor kappa-B kinase subunit beta	406	SII
Q9Z1X4	Interleukin enhancer-binding factor 3	110	SII
Q9D6R2	Isocitrate dehydrogenase [NAD] subunit alpha_ mitochondrial	71	SII
O54983	Ketimine reductase mu-crystallin	207	SII
P28738	Kinesin heavy chain isoform 5C	120	SII
O88447	Kinesin light chain 1	74	SII
L0N7N1	Kinesin-like protein KIF14	139	SII
Q60575	Kinesin-like protein KIF1B	29	SII
B1B1A0	Lethal(3)malignant brain tumor-like protein 4	85	SII
P60469	Liprin-alpha-3	44	SII
Q8C8U0	Liprin-beta-1	99	SII
Q61790	Lymphocyte activation gene 3 protein	101	SII
Q924M7	Mannose-6-phosphate isomerase	130	SII
Q3U435	Matrix metalloproteinase-25	89	SII
Q5HZI1	Microtubule-associated tumor suppressor 1 homolog	27	SII
Q9CW42	Mitochondrial amidoxime-reducing component 1	122	SII
Q9CXT8	Mitochondrial-processing peptidase subunit beta	145	SII
Q920G8	Mitoferrin-1	77	SII
P97820	Mitogen-activated protein kinase kinase kinase kinase 4	115	SII
Q8VCM2	NADPH oxidase organizer 1	145	SII
Q61043	Ninein	47	SII
Q6PIJ4	Nuclear factor related to kappa-B-binding protein	72	SII
Q99MH5	Nucleoside diphosphate kinase homolog 5	253	SII
Q9D478	Outer dense fiber protein 2-like	85	SII
P17742	Peptidyl-prolyl cis-trans isomerase A	263	SII
Q62009	Periostin	45	SII
P15331	Peripherin	45	SII
P35700	Peroxiredoxin-1	112	SII
A6H619	PHD and RING finger domain-containing protein 1	63	SII
Q8VEM8	Phosphate carrier protein_ mitochondrial	298	SII
P70296	Phosphatidylethanolamine-binding protein 1	131	SII
O70167	Phosphatidylinositol 4-phosphate 3-kinase C2 domain-containing subunit gamma	180	SII
Q9DBJ1	Phosphoglycerate mutase 1	150	SII
Q08481	Platelet endothelial cell adhesion molecule	143	SII
Q80UG2	Plexin-A4	41	SII
Q64028	Polyhomeotic-like protein 1	68	SII
Q3TVI8	Pre-B-cell leukemia transcription factor- interacting protein 1	89	SII
Q8C8T8	Pre-rRNA-processing protein TSR2 homolog	91	SII

Q62083	PRKCA-binding protein	115	SII
P54823	Probable ATP-dependent RNA helicase DDX6	90	SII
P57774	Pro-neuropeptide Y	135	SII
O70374	Protein CBFA2T2	50	SII
Q9DAF3	Protein DDI1 homolog 1	42	SII
Q9D0F3	Protein ERGIC-53	87	SII
Q80TL7	Protein MON2 homolog	41	SII
O88286	Protein Wiz	177	SII
Q8BQP8	Rab11 family-interacting protein 4	81	SII
Q62172	RalA-binding protein 1	85	SII
Q64487	Receptor-type tyrosine-protein phosphatase delta	140	SII
Q8CGE9	Regulator of G-protein signaling 12	162	SII
Q3UIA2	Rho GTPase-activating protein 17	55	SII
P61588	Rho-related GTP-binding protein RhoE	214	SII
Q64518	Sarcoplasmic/endoplasmic reticulum calcium ATPase 3	52	SII
Q60988	SCL-interrupting locus protein homolog	160	SII
Q3V129	Serine/threonine-protein kinase ULK4	54	SII
Q9EQY0	Serine/threonine-protein kinase/endo ribonuclease IRE1	143	SII
Q6PD03	Serine/threonine-protein phosphatase 2A 56 kDa regulatory subunit alpha isoform	161	SII
Q9CQR6	Serine/threonine-protein phosphatase 6 catalytic subunit	73	SII
Q8CIE0	Serpin A11	167	SII
Q923I7	Sodium/glucose cotransporter 2	110	SII
Q9Z0E8	Solute carrier family 22 member 5	69	SII
Q91WM1	Spermatid perinuclear RNA-binding protein	41	SII
Q80TF6	StAR-related lipid transfer protein 9	53	SII
Q8VIM6	Stereocilin	403	SII
P08228	Superoxide dismutase [Cu-Zn]	206	SII
Q8K4L3	Supervillin	159	SII
Q8CHC4	Synaptojanin-1	92	SII
Q91YE8	Synaptopodin-2	97	SII
Q8R1Q0	Syntaxin-19	274	SII
Q9D6E4	Tetratricopeptide repeat protein 9B	385	SII
Q3U0M1	Trafficking protein particle complex subunit 9	68	SII
Q924A0	Transcription factor 7-like 2	80	SII
Q8BRH0	Transmembrane and TPR repeat-containing protein 3	114	SII
Q9ERP3	Tripartite motif-containing protein 54	66	SII
P05214	Tubulin alpha-3 chain	195	SII
P68368	Tubulin alpha-4A chain	195	SII
Q9JJZ2	Tubulin alpha-8 chain	195	SII
Q7TMM9	Tubulin beta-2A chain	548	SII
Q9CWF2	Tubulin beta-2B chain	539	SII
P99024	Tubulin beta-5 chain	542	SII
Q922F4	Tubulin beta-6 chain	57	SII
Q9R1K7	Tubulin delta chain	127	SII
Q61333	Tumor necrosis factor alpha-induced protein 2	188	SII
P48025	Tyrosine-protein kinase SYK	97	SII
Q80WC1	Ubinuclein-2	59	SII
Q9CWU6	Ubiquinol-cytochrome-c reductase complex assembly factor 1	422	SII
Q9ES63	Ubiquitin carboxyl-terminal hydrolase 29	87	SII
Q6P5E4	UDP-glucose:glycoprotein glucosyltransferase1	292	SII

Q9DAD0	Uncharacterized protein C1orf194 homolog	111	SII
Q99104	Unconventional myosin-Va	66	SII
Q3TLD5	Unconventional prefoldin RPB5 interactor	115	SII
P51163	Uroporphyrinogen-III synthase	161	SII
Q91ZJ5	UTP--glucose-1-phosphate uridylyltransferase	116	SII
P59016	Vacuolar protein sorting-associated protein33B	119	SII
P29533	Vascular cell adhesion protein 1	62	SII
P20152	Vimentin	119	SII
Q60932	Voltage-dependent anion-selective channel protein 1	155	SII
Q99KC8	von Willebrand factor A domain-containing protein 5A	60	SII
Q5RJ54	Zinc finger and SCAN domain-containing protein 26	104	SII
Q6NS86	Zinc finger protein 366	53	SII
Q69Z99	Zinc finger protein 512	70	SII
Q8BGK2	[Protein ADP-ribosylarginine] hydrolase-like protein 1	280	RII
E9Q9A9	2'-5'-oligoadenylate synthase 2	85	RII
Q8QZS1	3-hydroxyisobutyryl-CoA hydrolase_ mitochondrial	115	RII
Q32Q92	Acyl-coenzyme A thioesterase 6	51	RII
B7ZCC9	Adhesion G-protein coupled receptor G4	84	RII
P61208	ADP-ribosylation factor-like protein 4C	222	RII
Q6P068	ADP-ribosylation factor-like protein 5C	69	RII
Q60604	Adseverin	51	RII
Q9QZQ1	Afadin	111	RII
P29699	Alpha-2-HS-glycoprotein	175	RII
Q8CFA2	Aminomethyltransferase_ mitochondrial	185	RII
Q9DBR4	Amyloid beta A4 precursor protein-binding family B member 2	227	RII
Q5F259	Ankyrin repeat domain-containing protein 13B	62	RII
Q80VM7	Ankyrin repeat domain-containing protein 24	214	RII
Q8BZW2	Ankyrin repeat domain-containing protein SOWAHB	75	RII
Q91YI0	Argininosuccinate lyase	90	RII
Q8K363	ATP-dependent RNA helicase DDX18	84	RII
Q6ZPL9	ATP-dependent RNA helicase DDX55	159	RII
Q80W49	Beta/gamma crystallin domain-containing protein 3	42	RII
O88428	Bifunctional 3'-phosphoadenosine 5'- phosphosulfate synthase 2	80	RII
B2RQC6	CAD protein	70	RII
Q8VHF2	Cadherin-related family member 5	107	RII
Q6Q473	Calcium-activated chloride channel regulator4A	56	RII
A2AHC3	Calmodulin-regulated spectrin-associated protein 1	64	RII
Q0VEJ0	Centrosomal protein of 76 kDa	65	RII
Q03059	Choline O-acetyltransferase	41	RII
O35218	Cleavage and polyadenylation specificity factor subunit 2	69	RII
Q8CDI7	Coiled-coil domain-containing protein 150	45	RII
Q8CE13	Coiled-coil domain-containing protein 17	55	RII
Q80X19	Collagen alpha-1(XIV) chain	74	RII
P03953	Complement factor D	304	RII
Q9CWX2	Complex I intermediate-associated protein 30_ mitochondrial	59	RII
Q8BLF2	Cyclin-dependent kinase-like 3	360	RII
Q99KY4	Cyclin-G-associated kinase	166	RII
Q9Z1J3	Cysteine desulfurase_ mitochondrial	90	RII
Q9CZ13	Cytochrome b-c1 complex subunit 1_ mitochondrial	95	RII
Q8K3G9	DCC-interacting protein 13-beta	79	RII
Q8CDG3	Deubiquitinating protein VCIP135	77	RII

P97427	Dihydropyrimidinase-related protein 1	136	RII
Q9D8U7	DTW domain-containing protein 1	81	RII
Q3V0Q1	Dynein heavy chain 12_ axonemal	108	RII
Q8C863	E3 ubiquitin-protein ligase Itchy	67	RII
O88196	E3 ubiquitin-protein ligase TTC3	109	RII
P55772	Ectonucleoside triphosphate diphosphohydrolase 1	78	RII
Q6R2P8	Endonuclease 8-like 2	98	RII
Q9CR89	Endoplasmic reticulum-Golgi intermediate compartment protein 2 O	258	RII
Q5D1E7	Endoribonuclease ZC3H12A	80	RII
Q3TLP5	Enoyl-CoA hydratase domain-containing protein 2_ mitochondrial	550	RII
O35393	Ephrin-B3	149	RII
Q924P3	Epididymal-specific lipocalin-8	148	RII
Q80VP1	Epsin-1	107	RII
Q8C7X2	ER membrane protein complex subunit 1	101	RII
P47753	F-actin-capping protein subunit alpha-1	159	RII
P47757	F-actin-capping protein subunit beta	95	RII
P11276	Fibronectin	150	RII
Q91Y97	Fructose-bisphosphate aldolase B	78	RII
Q571F8	Glutaminase liver isoform_ mitochondrial	94	RII
Q8BKV1	Glypican-2	211	RII
Q8VEF1	GRAM domain-containing protein 1A	98	RII
Q80TI0	GRAM domain-containing protein 1B	113	RII
P48722	Heat shock 70 kDa protein 4L	75	RII
A2AJ76	Hemicentin-2	413	RII
P79457	Histone demethylase UTY	42	RII
Q80W88	Homeobox and leucine zipper protein Homez	125	RII
Q08890	Iduronate 2-sulfatase	158	RII
Q91VK4	Integral membrane protein 2C	108	RII
Q9WVP9	Interferon-induced GTP-binding protein Mx2	210	RII
Q80XH2	Interphotoreceptor matrix proteoglycan 2	71	RII
Q6VH22	Intraflagellar transport protein 172 homolog	53	RII
Q8BFQ9	Kelch-like protein 42	137	RII
Q3V300	Kinesin-like protein KIF22	118	RII
Q3TJ91	Lethal(2) giant larvae protein homolog 2	193	RII
P62046	Leucine-rich repeat and calponin homology domain-containing protein 1	56	RII
Q8C0R9	Leucine-rich repeat and death domain- containing protein 1	99	RII
Q6P1C6	Leucine-rich repeats and immunoglobulin-like domains protein 3	88	RII
Q5SUF2	Luc7-like protein 3	135	RII
P27782	Lymphoid enhancer-binding factor 1	156	RII
Q8CAQ8	MICOS complex subunit Mic60	106	RII
P70218	Mitogen-activated protein kinase kinase kinase kinase 1	78	RII
Q61006	Muscle_ skeletal receptor tyrosine-protein kinase	57	RII
Q6NZR2	Myb/SANT-like DNA-binding domain- containing protein 2	59	RII
P11247	Myeloperoxidase	308	RII
Q8CI43	Myosin light chain 6B	74	RII
Q61941	NAD(P) transhydrogenase_ mitochondrial	70	RII
Q91YT0	NADH dehydrogenase [ubiquinone] flavoprotein 1_ mitochondrial	220	RII
P21661	Neuroendocrine convertase 2	111	RII
P49117	Nuclear receptor subfamily 2 group C member2	71	RII
Q8CH40	Nucleoside diphosphate-linked moiety X motif6	90	RII
Q80UF9	Otopetrin-3	59	RII

A3KGV1	Outer dense fiber protein 2	101	RII
P41593	Parathyroid hormone/parathyroid hormone- related peptide receptor	66	RII
Q9EPL9	Peroxisomal acyl-coenzyme A oxidase 3	269	RII
Q5SPL2	PHD finger protein 12	375	RII
Q8BLG0	PHD finger protein 20	65	RII
P26262	Plasma kallikrein	50	RII
P70458	Plasma serine protease inhibitor	106	RII
Q8CDU6	Probable E3 ubiquitin-protein ligase HECTD2	339	RII
Q6PAV2	Probable E3 ubiquitin-protein ligase HERC4	53	RII
Q9CR73	Proline-rich nuclear receptor coactivator 2	281	RII
P70403	Protein CASP	109	RII
Q8VE88	Protein FAM114A2	110	RII
Q8C627	Protein FAM221B	113	RII
Q6NZK5	Protein hinderin	107	RII
Q5DTZ0	Protein NYNRIN	48	RII
O35595	Protein patched homolog 2	69	RII
P97352	Protein S100-A13	190	RII
Q91XY4	Protocadherin gamma-A4	92	RII
Q3UNZ8	Quinone oxidoreductase-like protein 2	129	RII
Q60695	Ral guanine nucleotide dissociation stimulator- like 1	83	RII
Q8C2K5	RAS protein activator like-3	90	RII
Q9CX84	Regulator of G-protein signaling 19	131	RII
Q8K4Q0	Regulatory-associated protein of mTOR	120	RII
Q91YE7	RNA-binding protein 5	248	RII
Q8BX22	Sal-like protein 4	131	RII
P42208	Septin-2	255	RII
P98083	SHC-transforming protein 1	117	RII
P42230	Signal transducer and activator of transcription5A	157	RII
Q8R3P9	SMC5-SMC6 complex localization factor protein 1	77	RII
Q8C0X8	Sperm motility kinase X	71	RII
Q8BWB1	Synaptopodin 2-like protein	100	RII
Q9JKD8	T-box transcription factor TBX21	81	RII
P80318	T-complex protein 1 subunit gamma	90	RII
Q8K1H7	T-complex protein 11-like protein 2	55	RII
Q3URQ0	Testis-expressed protein 10	54	RII
Q61286	Transcription factor 12	235	RII
Q9EPK8	Transient receptor potential cation channel subfamily V member 4	96	RII
Q80W04	Transmembrane and coiled-coil domains protein 2	99	RII
Q6GQT5	Transmembrane protein 151A	125	RII
Q01887	Tyrosine-protein kinase RYK	183	RII
Q99NB8	Ubiquilin-4	102	RII
Q8C2S0	Ubiquitin carboxyl-terminal hydrolase 44	69	RII
O08759	Ubiquitin-protein ligase E3A	118	RII
Q8CE97	Uncharacterized protein C15orf62 homolog_ mitochondrial	194	RII
Q8C456	WD repeat-containing and planar cell polarity effector protein fritz homolog	451	RII
Q8BGF3	WD repeat-containing protein 92	398	RII
Q6NZF1	Zinc finger CCCH domain-containing protein11A	138	RII
O88532	Zinc finger RNA-binding protein	102	RII
Q60738	Zinc transporter 1	65	RII

[a]Identification is based on proteins ID from UniProt protein database, reviewed only (http://www.uniprot.org/).
[b]Proteins with expression significantly altered are organized according to the ratio.

*Indicates unique proteins in alphabetical order.

Table S6. Proteins with expression significantly altered in the gastrocnemius of SIII (A/J, water containing 50 ppm F, exercise) and RIII (129P3/J, water containing 50 ppm F, exercise) mice

[a]Acession number	Protein name	PLGS Score	[b]*Ratio* SIII: RIII
P32848	Parvalbumin alpha	6797	7.10
P97457	Myosin regulatory light chain 2_ skeletal muscle isoform	7435	5.05
P21107	Tropomyosin alpha-3 chain	262	4.53
Q64467	Glyceraldehyde-3-phosphate dehydrogenase_ testis-specific	74	3.53
P02104	Hemoglobin subunit epsilon-Y2	2664	3.49
P16858	Glyceraldehyde-3-phosphate dehydrogenase	14569	3.19
O09165	Calsequestrin-1	1129	2.92
Q3UHK3	Protein GREB1	121	2.89
Q9DBJ1	Phosphoglycerate mutase 1	54	2.86
P02089	Hemoglobin subunit beta-2	2664	2.72
P11404	Fatty acid-binding protein_ heart	178	2.59
Q60611	DNA-binding protein SATB1	218	2.56
Q91VW5	Golgin subfamily A member 4	227	2.53
P02088	Hemoglobin subunit beta-1	3946	2.53
P13412	Troponin I_ fast skeletal muscle	2648	2.53
P07310	Creatine kinase M-type	16395	2.48
P13541	Myosin-3	2675	2.29
Q5SX39	Myosin-4	18478	2.27
A2AQP0	Myosin-7B	428	2.23
P57780	Alpha-actinin-4	80	2.14
P05977	Myosin light chain 1/3_ skeletal muscle isoform	22171	2.10
Q91Z83	Myosin-7	5146	2.05
P70402	Myosin-binding protein H	203	2.01
Q5SX40	Myosin-1	13910	1.99
P17183	Gamma-enolase	806	1.97
Q7TQ48	Sarcalumenin	41	1.95
P52480	Pyruvate kinase PKM	4168	1.92
P17751	Triosephosphate isomerase	3267	1.88
Q9R0Y5	Adenylate kinase isoenzyme 1	2915	1.84
O70250	Phosphoglycerate mutase 2	984	1.80
P08249	Malate dehydrogenase_ mitochondrial	475	1.79
P01942	Hemoglobin subunit alpha	9144	1.77
P53657	Pyruvate kinase PKLR	737	1.77
P17182	Alpha-enolase	2341	1.75
Q8BFZ3	Beta-actin-like protein 2	14800	1.75
Q3TJD7	PDZ and LIM domain protein 7	268	1.73
P60710	Actin_ cytoplasmic 1	49058	1.72
P09041	Phosphoglycerate kinase 2	1520	1.72
P21550	Beta-enolase	4470	1.70
O08638	Myosin-11	255	1.68
P63260	Actin_ cytoplasmic 2	49058	1.67
P47857	ATP-dependent 6-phosphofructokinase_	165	1.67
P0DP28	Calmodulin-3	389	1.67
P68033	Actin_ alpha cardiac muscle 1	66606	1.65
P31001	Desmin	273	1.65

P04247	Myoglobin	381	1.65
Q02566	Myosin-6	3955	1.65
P09411	Phosphoglycerate kinase 1	1615	1.65
Q9JI91	Alpha-actinin-2	177	1.63
P0DP27	Calmodulin-2	389	1.63
P58774	Tropomyosin beta chain	6233	1.63
P62737	Actin_ aortic smooth muscle	61441	1.62
P13542	Myosin-8	8481	1.62
Q8VDD5	Myosin-9	255	1.62
P58771	Tropomyosin alpha-1 chain	10393	1.62
P68134	Actin_ alpha skeletal muscle	73343	1.60
P63268	Actin_ gamma-enteric smooth muscle	61339	1.60
P0DP26	Calmodulin-1	389	1.60
P06151	L-lactate dehydrogenase A chain	1760	1.55
O88990	Alpha-actinin-3	989	1.54
Q9D0F9	Phosphoglucomutase-1	80	1.54
Q5XKE0	Myosin-binding protein C_ fast-type	2097	1.52
P16125	L-lactate dehydrogenase B chain	309	1.51
P07724	Serum albumin	636	1.51
P09542	Myosin light chain 3	4350	1.49
Q7TPR4	Alpha-actinin-1	57	1.48
Q60605	Myosin light polypeptide 6	1016	1.48
P05064	Fructose-bisphosphate aldolase A	12552	1.46
P14152	Malate dehydrogenase_ cytoplasmic	202	1.43
P00342	L-lactate dehydrogenase C chain	455	1.42
P09541	Myosin light chain 4	5093	1.34
Q99LX0	Protein DJ-1	503	1.34
Q62234	Myomesin-1	80	1.32
Q6URW6	Myosin-14	258	1.31
Q9QZ47	Troponin T_ fast skeletal muscle	1180	1.30
Q99KI0	Aconitate hydratase_ mitochondrial	100	1.28
Q9JKS4	LIM domain-binding protein 3	618	1.28
Q61879	Myosin-10	256	1.26
O55143	Sarcoplasmic/endoplasmic reticulum calcium ATPase 2	244	1.25
P16015	Carbonic anhydrase 3	1180	1.17
Q6P8J7	Creatine kinase S-type_ mitochondrial	300	1.17
P20801	Troponin C_ skeletal muscle	1693	1.13
P56480	ATP synthase subunit beta_ mitochondrial	1511	1.12
Q9WUB3	Glycogen phosphorylase_ muscle form	4418	1.09
Q8R429	Sarcoplasmic/endoplasmic reticulum calcium ATPase 1	946	1.07
Q5SPL2	PHD finger protein 12	495	0.73
Q3UH93	Plexin-D1	95	0.50
Q80TM9	Nischarin	292	0.35
Q8CJH3	Plexin-B1	169	0.30
Q9CZU6	Citrate synthase_ mitochondrial	51	0.27
Q8R4I1	Ataxin-7	193	0.21
P62259	14-3-3 protein epsilon	81	SIII*
O88986	2-amino-3-ketobutyrate coenzyme A ligase_ mitochondrial	55	SIII
O08756	3-hydroxyacyl-CoA dehydrogenase type-2	73	SIII
Q921H8	3-ketoacyl-CoA thiolase A_ peroxisomal	114	SIII
Q8VCH0	3-ketoacyl-CoA thiolase B_ peroxisomal	114	SIII

P20029	78 kDa glucose-regulated protein	111	SIII
Q6Q2Z6	Acyl-coenzyme A thioesterase 5	71	SIII
Q9ESW4	Acylglycerol kinase_ mitochondrial	50	SIII
Q61315	Adenomatous polyposis coli protein	73	SIII
P45376	Aldose reductase	98	SIII
Q9QYC0	Alpha-adducin	80	SIII
P46660	Alpha-internexin	58	SIII
Q9D4H4	Angiomotin-like protein 1	45	SIII
Q9D0I9	Arginine--tRNA ligase_ cytoplasmic	46	SIII
Q91V24	ATP-binding cassette sub-family A member 7	41	SIII
P12382	ATP-dependent 6-phosphofructokinase_ liver type	71	SIII
P52963	Band 4.1-like protein 4A	50	SIII
P41183	B-cell lymphoma 6 protein homolog	69	SIII
P97929	Breast cancer type 2 susceptibility protein homolog	66	SIII
P97291	Cadherin-8	71	SIII
Q9JKL5	Calcineurin B homologous protein 3	130	SIII
P58660	Caspase recruitment domain-containing protein 10	42	SIII
Q6A065	Centrosomal protein of 170 kDa	73	SIII
Q9ESN9	C-Jun-amino-terminal kinase-interacting protein 3	107	SIII
Q9QXK3	Coatomer subunit gamma-2	47	SIII
Q6NS45	Coiled-coil domain-containing protein 66	51	SIII
Q4QRL3	Coiled-coil domain-containing protein 88B	54	SIII
Q9DBT3	Coiled-coil domain-containing protein 97	97	SIII
P11087	Collagen alpha-1(I) chain	40	SIII
Q01149	Collagen alpha-2(I) chain	36	SIII
Q9CZ13	Cytochrome b-c1 complex subunit 1_ mitochondrial	170	SIII
P12787	Cytochrome c oxidase subunit 5A_ mitochondrial	122	SIII
P62897	Cytochrome c_ somatic	375	SIII
Q91YE9	Cytosolic 5'-nucleotidase 1B	42	SIII
Q91VU6	DDB1- and CUL4-associated factor 11	72	SIII
Q8R1A4	Dedicator of cytokinesis protein 7	28	SIII
Q8VC56	E3 ubiquitin-protein ligase RNF8	49	SIII
Q6P5F9	Exportin-1	86	SIII
P47754	F-actin-capping protein subunit alpha-2	101	SIII
Q61553	Fascin	118	SIII
Q8VHX6	Filamin-C	28	SIII
O08917	Flotillin-1	70	SIII
Q9WTJ4	Flt3-interacting zinc finger protein 1	733	SIII
Q99JB6	Forkhead box protein P3	55	SIII
Q810T2	G2/mitotic-specific cyclin-B3	52	SIII
Q5SNZ0	Girdin	177	SIII
P03995	Glial fibrillary acidic protein	59	SIII
C0HKE1	Histone H2A type 1-B	691	SIII
C0HKE2	Histone H2A type 1-C	691	SIII
C0HKE3	Histone H2A type 1-D	691	SIII
C0HKE4	Histone H2A type 1-E	691	SIII
Q8CGP5	Histone H2A type 1-F	691	SIII
C0HKE5	Histone H2A type 1-G	691	SIII
Q8CGP6	Histone H2A type 1-H	691	SIII
C0HKE6	Histone H2A type 1-I	691	SIII
Q8CGP7	Histone H2A type 1-K	691	SIII

C0HKE7	Histone H2A type 1-N	691	SIII
C0HKE8	Histone H2A type 1-O	691	SIII
C0HKE9	Histone H2A type 1-P	691	SIII
Q6GSS7	Histone H2A type 2-A	691	SIII
Q64522	Histone H2A type 2-B	201	SIII
Q64523	Histone H2A type 2-C	691	SIII
Q8BFU2	Histone H2A type 3	691	SIII
Q8R1M2	Histone H2A.J	691	SIII
Q3THW5	Histone H2A.V	201	SIII
P0C0S6	Histone H2A.Z	201	SIII
P27661	Histone H2AX	201	SIII
P97443	Histone-lysine N-methyltransferase Smyd1	98	SIII
P18533	Ig heavy chain V region 733	108	SIII
Q3USB7	Inactive phospholipase C-like protein 1	353	SIII
Q99PW8	Kinesin-like protein KIF17	36	SIII
Q7TNC6	Kinesin-like protein KIF26B O	38	SIII
Q8CDB0	MAP kinase-interacting serine/threonine- protein kinase 2	56	SIII
P15089	Mast cell carboxypeptidase A	48	SIII
Q9JI70	McKusick-Kaufman/Bardet-Biedl syndromes putative chaperonin	54	SIII
A2AG06	Meiosis-specific coiled-coil domain- containing protein MEIOC	36	SIII
Q8BI84	Melanoma inhibitory activity protein 3	107	SIII
Q9EQ20	Methylmalonate-semialdehyde dehydrogenase[acylating]_ mitochondrial	78	SIII
Q9JIF9	Myotilin	133	SIII
Q3TKR3	NACHT_ LRR and PYD domains-containing protein 4C	31	SIII
P19246	Neurofilament heavy polypeptide	45	SIII
P08551	Neurofilament light polypeptide	45	SIII
P08553	Neurofilament medium polypeptide	45	SIII
Q9WU42	Nuclear receptor corepressor 2	92	SIII
O88708	Origin recognition complex subunit 4	43	SIII
B9EJ86	Oxysterol-binding protein-related protein 8	39	SIII
P48725	Pericentrin	78	SIII
P15331	Peripherin	43	SIII
Q8VD65	Phosphoinositide 3-kinase regulatory subunit 4	42	SIII
Q99KP6	Pre-mRNA-processing factor 19	117	SIII
P54823	Probable ATP-dependent RNA helicase DDX6	88	SIII
Q8CDU6	Probable E3 ubiquitin-protein ligase HECTD2	191	SIII
Q812A5	Proline-rich protein 5	68	SIII
Q9DAU1	Protein canopy homolog 3	167	SIII
Q9DAF3	Protein DDI1 homolog 1	48	SIII
Q8R100	Protein FAM26E	95	SIII
Q8BZ32	Putative Polycomb group protein ASXL2	48	SIII
Q69ZJ7	RAB6A-GEF complex partner protein 1	31	SIII
Q5FWH6	Rho guanine nucleotide exchange factor 15	37	SIII
Q8C2Q3	RNA-binding protein 14	127	SIII
Q9WTM3	Semaphorin-6C	179	SIII
Q9DBP0	Sodium-dependent phosphate transport protein2B	173	SIII
Q922B9	Sperm-specific antigen 2 homolog	38	SIII
G3X912	SprT-like domain-containing protein Spartan	102	SIII
Q6PE84	Stomatin-like protein 3	133	SIII
P08228	Superoxide dismutase [Cu-Zn]	236	SIII

P70327	T-box transcription factor TBX6	66	SIII
P20108	Thioredoxin-dependent peroxide reductase_ mitochondrial	83	SIII
Q61286	Transcription factor 12	96	SIII
Q9WUZ5	Troponin I_ slow skeletal muscle	116	SIII
Q6QR59	TRPM8 channel-associated factor 3	54	SIII
P68369	Tubulin alpha-1A chain	107	SIII
P05213	Tubulin alpha-1B chain	107	SIII
P68373	Tubulin alpha-1C chain	107	SIII
P05214	Tubulin alpha-3 chain	35	SIII
P68368	Tubulin alpha-4A chain	44	SIII
Q06806	Tyrosine-protein kinase receptor Tie-1	49	SIII
Q01887	Tyrosine-protein kinase RYK	408	SIII
Q91WQ3	Tyrosine--tRNA ligase_ cytoplasmic	71	SIII
Q8C7R4	Ubiquitin-like modifier-activating enzyme 6	43	SIII
O08759	Ubiquitin-protein ligase E3A	100	SIII
Q3TQQ9	Uncharacterized protein C1orf112 homolog	37	SIII
Q8CC96	Uncharacterized protein C6orf222 homolog	48	SIII
Q9D454	Uncharacterized protein CXorf49 homolog	79	SIII
A2AAE1	Uncharacterized protein KIAA1109	24	SIII
Q9QY06	Unconventional myosin-IXb	32	SIII
P20152	Vimentin	71	SIII
Q60932	Voltage-dependent anion-selective channel protein 1	435	SIII
Q3UR50	von Willebrand factor A domain-containing protein 5B2	86	SIII
Q5F293	Zinc finger and BTB domain-containing protein 4	71	SIII
Q61464	Zinc finger protein 638	90	SIII
Q921S7	39S ribosomal protein L37_ mitochondrial	74	RIII
Q9D404	3-oxoacyl-[acyl-carrier-protein] synthase_ mitochondrial	56	RIII
Q9JII1	72 kDa inositol polyphosphate 5-phosphatase	74	RIII
Q9QY83	Actin-like protein 7B	129	RIII
Q8BK64	Activator of 90 kDa heat shock protein ATPase homolog 1	58	RIII
Q9QZQ1	Afadin	101	RIII
Q9DBR4	Amyloid beta A4 precursor protein-binding family B member 2	155	RIII
Q8BZ05	Arf-GAP with Rho-GAP domain_ ANK repeat and PH domain-containing protein 2	91	RIII
Q8R3P0	Aspartoacylase	107	RIII
Q61137	Astrotactin-1	62	RIII
Q9D3D9	ATP synthase subunit delta_ mitochondrial	285	RIII
O88738	Baculoviral IAP repeat-containing protein 6	75	RIII
O88428	Bifunctional 3'-phosphoadenosine 5'- phosphosulfate synthase 2	84	RIII
Q9CWJ9	Bifunctional purine biosynthesis protein PURH	230	RIII
Q6DFY8	BMP/retinoic acid-inducible neural-specific protein 2	97	RIII
Q499E0	BMP/retinoic acid-inducible neural-specific protein 3	158	RIII
Q52KB6	C2 domain-containing protein 3	81	RIII
P08607	C4b-binding protein	84	RIII
Q91ZI0	Cadherin EGF LAG seven-pass G-type receptor 3	76	RIII
Q7TQK5	Coiled-coil domain-containing protein 93	82	RIII
Q8R1U1	Conserved oligomeric Golgi complex subunit4	86	RIII
Q8BLF2	Cyclin-dependent kinase-like 3	80	RIII
Q7TMW6	Cytosolic Fe-S cluster assembly factor NARFL	89	RIII
Q8K3G9	DCC-interacting protein 13-beta	182	RIII
Q8N7N5	DDB1- and CUL4-associated factor 8	121	RIII
O55111	Desmoglein-2	61	RIII

Q811D0	Disks large homolog 1	41	RIII
P0C6F1	Dynein heavy chain 2_ axonemal	32	RIII
Q4U2R1	E3 ubiquitin-protein ligase HERC2	416	RIII
Q9ERK4	Exportin-2	118	RIII
A2A870	Fas-binding factor 1	359	RIII
P42128	Forkhead box protein K1	75	RIII
Q8K284	General transcription factor 3C polypeptide 1	152	RIII
Q9WUU9	Germinal-center associated nuclear protein	96	RIII
Q9R257	Heme-binding protein 1	83	RIII
Q9JHU9	Inositol-3-phosphate synthase 1	83	RIII
P54071	Isocitrate dehydrogenase [NADP]_ mitochondrial	212	RIII
Q8BIJ6	Isoleucine--tRNA ligase_ mitochondrial	78	RIII
Q69ZK5	Kelch-like protein 14	106	RIII
Q497I4	Keratin_ type I cuticular Ha5	65	RIII
Q99M74	Keratin_ type II cuticular Hb2	88	RIII
Q61097	Kinase suppressor of Ras 1	448	RIII
Q91W40	Kinesin light chain 3	63	RIII
Q8K1S5	Krueppel-like factor 11	365	RIII
A2AHG0	Leucine zipper putative tumor suppressor 3	45	RIII
Q3UZ18	Little elongation complex subunit 2 O	32	RIII
Q99MN1	Lysine--tRNA ligase	57	RIII
Q8CAQ8	MICOS complex subunit Mic60	216	RIII
Q9JM52	Misshapen-like kinase 1	86	RIII
Q9Z2I0	Mitochondrial proton/calcium exchanger protein	72	RIII
Q07174	Mitogen-activated protein kinase kinase kinase8	121	RIII
Q91YD3	mRNA-decapping enzyme 1A	68	RIII
Q9Z2C4	Myotubularin-related protein 1	86	RIII
Q61578	NADPH:adrenodoxin oxidoreductase_ mitochondrial	56	RIII
E9Q7X7	Neurexin II	61	RIII
B0F2B4	Neuroligin 4-like	101	RIII
Q99K10	Neuroligin-1	107	RIII
Q69ZK9	Neuroligin-2	101	RIII
Q02780	Nuclear factor 1 A-type	123	RIII
P70255	Nuclear factor 1 C-type	123	RIII
Q6ZQH8	Nucleoporin NUP188 homolog	151	RIII
Q9DCM7	Nucleus accumbens-associated protein 2	229	RIII
O70209	PDZ and LIM domain protein 3	169	RIII
Q8CI51	PDZ and LIM domain protein 5	460	RIII
Q3TKY6	Peptidyl-prolyl cis-trans isomerase CWC27 homolog	145	RIII
P70296	Phosphatidylethanolamine-binding protein 1	227	RIII
Q7TQG1	Pleckstrin homology domain-containing family A member 6	424	RIII
Q8JZX3	POC1 centriolar protein homolog A	108	RIII
Q91Z31	Polypyrimidine tract-binding protein 2	65	RIII
Q3URV1	Protein broad-minded	101	RIII
Q8K2Y3	Protein eva-1 homolog B	281	RIII
Q3UY90	Protein FAM198A	78	RIII
Q3HNM7	Protein inscuteable homolog	192	RIII
O35595	Protein patched homolog 2	166	RIII
Q80U72	Protein scribble homolog	284	RIII
Q4KUS2	Protein unc-13 homolog A	116	RIII
Q64455	Receptor-type tyrosine-protein phosphatase eta	103	RIII
Q8CGE9	Regulator of G-protein signaling 12	75	RIII
Q8K4Q0	Regulatory-associated protein of mTOR	111	RIII

Q64518	Sarcoplasmic/endoplasmic reticulum calcium ATPase 3	34	RIII
Q9WUN2	Serine/threonine-protein kinase TBK1	91	RIII
P97470	Serine/threonine-protein phosphatase 4 catalytic subunit	132	RIII
Q921I1	Serotransferrin	419	RIII
Q8BPQ7	Small G protein signaling modulator 1	107	RIII
Q8K4L3	Supervillin	57	RIII
Q9D818	Suppressor APC domain-containing protein 2	74	RIII
Q6A028	Switch-associated protein 70	63	RIII
Q8CHC4	Synaptojanin-1	188	RIII
Q91YE8	Synaptopodin-2	44	RIII
Q62288	Testican-1	92	RIII
Q2TV84	Transient receptor potential cation channel subfamily M member 1	292	RIII
Q9EPK8	Transient receptor potential cation channel subfamily V member 4	79	RIII
Q91VP7	Transmembrane protein 101	78	RIII
Q99PP6	Tripartite motif-containing protein 34A	90	RIII
Q9CWH5	tRNA (guanine(10)-N2)-methyltransferase homolog	185	RIII
Q9D0C4	tRNA (guanine(37)-N1)-methyltransferase	80	RIII
Q9R0M8	UDP-galactose translocator	257	RIII
E9Q035	Uncharacterized protein	122	RIII
F8VQB6	Unconventional myosin-X	136	RIII

[a]Identification is based on proteins ID from UniProt protein database, reviewed only (http://www.uniprot.org/).
[b]Proteins with expression significantly altered are organized according to the ratio.
*Indicates unique proteins in alphabetical order.
